# Microbial community structure and functional traits involved in the adaptation of culturable bacteria within the gut of amphipods from the deepest ocean

**DOI:** 10.1128/spectrum.00723-24

**Published:** 2024-12-10

**Authors:** Yukun Cui, Yu Xiao, Zhuo Wang, Paiyao Ji, Changhao Zhang, Yongqi Li, Jiasong Fang, Xi Yu

**Affiliations:** 1 Shanghai Engineering Research Center of Hadal Science and Technology, College of Oceanography and Ecological Science, Shanghai Ocean University, Shanghai, China; University of Mississippi, University, Mississippi, USA

**Keywords:** gut microbes, amphipods, Mariana Trench, genome, environmental adaptation

## Abstract

The Hadal Zone is acknowledged for its extreme environmental conditions, especially high hydrostatic pressures. The dominant scavengers in the Hadal Zone, Hadal amphipods, fulfill vital roles in the Hadal food web and ecological niches. However, research on the gut microbiota of amphipods related to ecological functions and environmental adaptation is still limited. Here, we used 16S rRNA sequencing technology and a culture-dependent method to analyze the composition of the gut microbiota in Amphipoda living in the Mariana Trench. A total of 16 bacterial genera were identified. Among them, Firmicutes and Proteobacteria were the predominant phyla. The adaptability of gut probiotics to the environment was investigated. *Pediococcus pentosaceus* XY62 was picked up as the representative strain to elucidate the ecological functions of gut microbes in amphipods. The ProBio database and the K-B agar diffusion method indicated that *P. pentosaceus* XY62 exhibited the highest probiotic activity compared with all other isolated strains. Specific metabolic pathways and transporter systems that contribute to a range of environmental adaptation strategies have been revealed by genomic analysis of *P. pentosaceus* XY62. The environmental response genes and a specialized KDP transport system allow it to adapt to the challenging conditions of the Hadal Zone. In addition, the presence of antibacterial compounds and antibiotic resistance genes, as well as the ability to form a biofilm, facilitated the successful colonization of *P. pentosaceus* XY62 in the gut environment.

**IMPORTANCE:**

Amphipods are widely distributed in the Hadal trenches, and the study of their gut microbes has garnered considerable scientific interest. Our research breaks away from traditional omics approaches, innovatively combining sequencing technologies with culture-dependent methods to analyze the gut microbiome structure of amphipods from the Mariana Trench. This not only complements the current omics-dominated field but also paves the way for future resource development of extreme microbes. Furthermore, by conducting genomic analyses and functional validations on a representative strain, we have uncovered its probiotic effects and strategies for adapting to extreme environments. This provides new insights into the theoretical study of the ecological functions of deep-sea bacteria. Overall, our findings offer a fresh perspective on the microbial community structure and environmental adaptation strategies of gut microorganisms in the Hadal Zone.

## INTRODUCTION

The Hadal Zone constitutes the deepest region of the ocean, typically referring to areas with a depth range of 6,000 to 11,000 meters. It only accounts for 1%–2% of the benthic area but encompasses the deepest 45% of the vertical depth gradient (1). It is almost entirely composed of trenches representing spatially disjoint environments, separated by shallower areas (2). The Hadal environment poses severe environmental challenges, as the trench endures low water temperatures (1–2°C), high hydrostatic pressure (>60 MPa), and restricted food resources (3–5). These factors create unfavorable living conditions within the trench. In recent years, advances in deep-sea sampling have revealed rich biological communities at the bottom of the trench (6–9). This has improved our understanding of the Hadal environment and sparked extensive interest in exploring the adaptive mechanisms of the distinct organisms residing in the Hadal habitat (10, 11).

With increasing depth, the abundance and biomass of organisms generally decrease (12, 13). Nonetheless, in recent years, abundant amphipod communities (Crustacea: Peracarida: Amphipoda) have been identified in numerous Hadal trenches, including the deepest zone (14–16). Amphipods were identified as the principal scavengers in the trench and are widely recognized as a critical component of the Hadal ecosystem (17). To overcome oligotrophic conditions, they have evolved specialized mandibles and efficient digestive systems, which allow them to consume large amounts of food in a short period and store the energy derived from high-energy sources, such as carrion, for later use (18–22). Therefore, they become central to studying nutrient cycling and environmental adaptation in the Hadal Zone (15). The gut microbiota plays a crucial probiotic role in defending against pathogens, boosting the immune system, and improving the host’s ability to adapt to the environment (23–25). Certain studies have begun to reveal how the gut microbiota support the environmental adaptability of Hadal Amphipoda. Zhang et al. revealed the differences in the structure and function of gut microbiota among conspecific and heterospecific amphipods from different trenches based on metagenomics, detailing the evolutionary strategies of the gut bacterium *Psychromonas* to adapt to the Hadal trench environment (26, 27). Meanwhile, Chan et al. uncovered various probiotic functions of gut microbes in deep-sea amphipods, including animal survival, animal growth, and immune protection (28, 29). These research findings provide important insights for a deeper understanding of the diversity and functions of gut microbiota in deep-sea amphipods. At present, the metagenomic method has been widely used, significantly enhancing our understanding of the symbiotic microorganisms of amphipods in the Hadal Zone. Although the culture-dependent method for studying gut microbes in amphipods is underutilized. Recently, Kusube M. et al. isolated a new species of Colwellia from amphipods inhabiting the Mariana Trench, which can grow at extremely high hydrostatic pressures (80–140 MPa) (30). This highlights the potential of using a culture-dependent method to explore the functions and adaptations of amphipod gut microbiota.

In this study, we utilized microbial culture technology and 16S rRNA sequencing to characterize the gut microbial structure and probiotic functional characteristics of *Hysterocrates gigas* located in the Mariana Trench. The combination of the two methods revealed different compositions of bacterial species in the gut of amphipods. Probiotic function analysis and the K-B test showed that *Pediococcus pentosaceus* exhibited significant potential probiotic activity among the isolated strains. *P. pentosaceus* XY62, the first isolated species of the genus *Pentococcus*, was selected as the representative strain. The genome annotation and comprehensive analysis of *P. pentosaceus* XY62 were conducted, revealing its unique genes and mechanisms related to environmental adaptation. Meanwhile, we observed the response of the KDP system to hydrostatic pressure for the first time. Concurrently, the isolation and characterization of secondary metabolites of *P. pentosaceus* XY62 were conducted to determine its probiotic properties and environmental adaptability.

## MATERIALS AND METHODS

### Sample collection and processing

**Fig 1:**
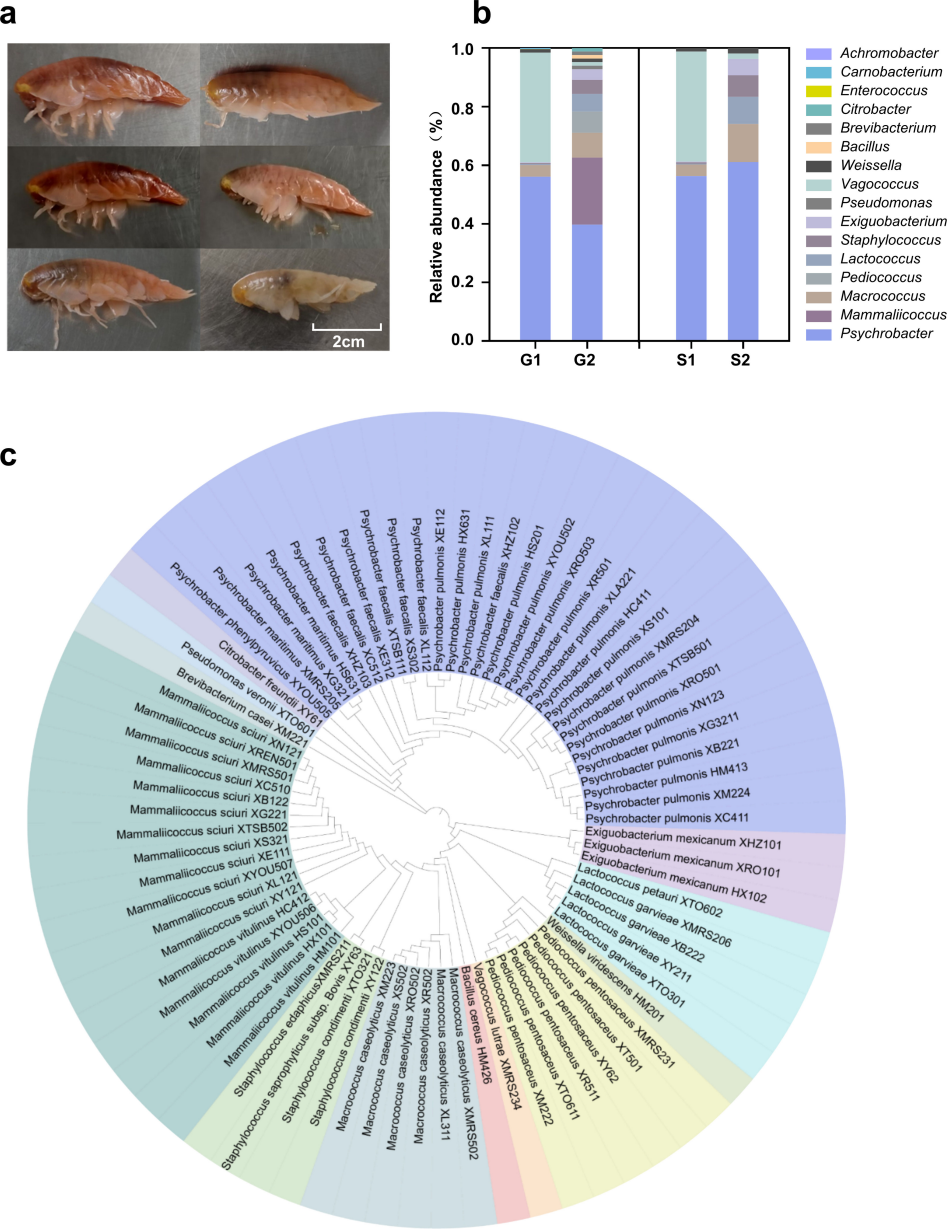
Characteristics of gut microbial community structure of amphipods. (**a**) Photo of six *H*. *gigas* individuals. (**b**) Gut bacterial community structure detected by 16S rRNA sequencing technology and culturing techniques (G1: Genus levels determined by 16S rRNA sequencing technology; G2: Genus levels determined by microbial culture technology; S1: Abundance of co-detected genera determined by 16S rRNA sequencing technology; and S2: Abundance of co-detected genera determined by microbial culture technology). GraphPad Prism 8.0.3. (**c**) Phylogenetic tree of 77 pure isolates based on genetic relationships. https://itol.embl.de/.

*H. gigas* specimens were collected from the Challenger Deep of the Mariana Trench in September 2021, using a macro-biological pressure-retaining sampler deployed from the “Explore I” research vessel during the TS-21 expeditions. Two sampling points were located at A (11°19.6098′N, 142°11.283′E) at a depth of 10,895 m and B (11°22.584′N, 142°35.3102′E) at a depth of 10,910 m, respectively (Fig. 1a; Fig. S1a). All samples (*n* = 6) were collected from the above two locations. The amphipods were immediately stored at −80°C upon collection. The samples were transferred to 4°C for thawing and rinsed thoroughly with distilled water (dd H_2_O). All amphipods were dissected using a sterile scalpel, and the gut tissues were carefully removed and preserved in 2 mL centrifuge tubes at 4°C.

### Gut bacteria screening and conservation

For bacterial isolation, the gut contents of the amphipods were diluted to 10^−2^, 10^−3^, and 10^−4^ with freshly prepared 3.4% sterile NaCl. Then, 200 µL of each dilution was plated on different types of isolation media. Briefly, 23 different media were used for strain screening, including Luria-Bertani Agar (LB), 2216E Agar, Potato Dextrose Agar (PDA), Yeast Extract Peptone Dextrose Medium (YPD), Brain Heart Infusion Agar (BHIA, Hopebio, Qingdao, China), Oatmeal Agar (OMA), Corn Meal Agar (CMA), Nutrient Agar (NA), etc. (Table S1). The plates were incubated at 20°C for 2–3 days, and visible colonies were picked and transferred to a new isolation medium for purification. The isolates were purified three times and then stored in 50% sterile glycerol at −80°C.

### DNA extraction and 16S rDNA sequencing of bacterial isolates

Genomic DNA was extracted using the Rapid Bacterial Genomic DNA Isolation Kit (Sangon, Shanghai, China) according to the manufacturer’s protocols. 16S rDNA was amplified using the universal bacterial primers 27F (5′-AGAGTTTGATCCTGGCTCAG-3′) and 1492R (5′-GGTTACCTTGTTACGACTT-3′). PCR reactions included 1 µL of genomic DNA template, 2 µL of primer 27F, 2 µL of primer 1492R, 20 µL of 2*× Accurate* Taq Master Mix (Accurate Biology, Hunan, China), and 25 µL of dd H_2_O. The PCR amplification procedure consisted of an initial denaturing step at 98°C for 2 min, followed by 30 cycles at 95°C for 15 s, 50°C for 30 s, and 72°C for 90 s. After sequencing by Azenta Life Science Co., Ltd. using the Applied Biosystems 3730 DNA Analyzer platform, the amplified 16S rDNA sequences were analyzed and identified against sequences in the NCBI database using the Blastn program. Phylogenetic trees were constructed using MEGA11 (31).

### 16S rRNA sequencing and operational taxonomic unit (OTU) analysis of amphipod gut bacteria

Genomic DNA was extracted from the amphipod’s gut contents using the E.Z.N.A. Soil DNA Kit (Omega Bio-Tek, Norcross, GA, United States). The 16S rRNA gene was amplified using the universal bacterial primers 338F (5′-ACTCCTACGGGAGGCAGCAG-3′) and 806R (5′-GGACTACHVGGGTWTCTAAT-3′) (29). Subsequently, it was extracted using the AxyPrep DNA Gel Extraction Kit (Axygen Biosciences, Union City, CA, USA) and quantified using the QuantFluor-ST blue fluorescence quantitative system (Promega, USA). The concentrations were determined according to the instrument’s manual of sequencing machines. The purified PCR products were processed on an Illumina MiSeq PE300 platform by Majorbio Bio-Pharm Technology Co. Ltd. (Shanghai, China). Non-repetitive sequences were extracted, and the single sequence without repeats was removed. Then, OTUs were clustered using UPARSE version 11, with a 97% similarity cutoff (32). Subsequently, representative sequences of OTUs were analyzed using RDP Classifier version 2.13, and the confidence threshold was 0.7 (33). Annotation and function prediction of gut probiotics were carried out based on the Probio Database (34).

### Genome assembly and genomic characteristics

*P. pentosaceus* XY62 was initially identified through 16S rDNA sequencing and cultured in liquid De Man, Rogosa and Sharp (MRS) medium for 3 days at 20°C for genomic DNA extraction. The Wizard Genomic DNA Purification Kit (Promega) was used to extract genomic DNA, following the manufacturer’s protocol. The sequencing work was completed by Majorbio Bio-Pharm Technology Co. Ltd. (Shanghai, China). Specifically, the whole genome was sequenced with the PacBio (Pacific Biosciences) and IIllumina HiSeq X Ten sequencing platforms. Genomic DNA was purified and collected to construct a genomic library. The optimal contigs were produced by assembling the Illumina sequencing data and quality-filtered using fastp v0.23.0 (35). The contigs were partially assembled and optimized to create scaffolds using SOAPdenovo2 (36). The assembly tool Unicycler was used to assemble the sequence into a ring and remove one of the overlapping sequences (37). Finally, the complete chromosome and plasmid sequences were obtained. NR, Swiss-Prot (38), Pfam (39), EggNOG (40), GO (41), and KEGG (42) were used to characterize the egg data of the sequenced genome (e-value <10^−5^).

### Comparative genome analysis

The genome of *P. pentosaceus* XY62 was compared with 23 complete genome sequences of the same species isolated from various sources, including plants, soil, tapioca, fermented dairy, sake mash, kimchi, shrimp, doenjang, soybean paste, and adult feces, obtained from NCBI. We utilized the Kyoto Encyclopedia of Genes and Genomes (KEGG) database and the Evolutionary Genealogy of Genes: Non-supervised Orthologous Groups (eggNOG) database to identify potential differences in gene functions. Environmental-related genes were annotated using the Rapid Annotation within the Subsystem Technology (RAST) database. The carbohydrate-active enzymes were predicted using the dbCAN3 Database with HMMER dbCAN (e-value <1e-15; coverage >0.35) (43). The metabolism gene clusters were predicted using the antiSMASH Database and the BAGEL4 Database.

### Quantitative real-time reverse transcription PCR (qRT-PCR) analysis

A set of qRT-PCR experiments were performed to assess the response of *P. pentosaceus* XY62 to different salinities (0%, 1%, 2%, and 3% NaCl), temperatures (4°C, 10°C, 20°C, and 28°C), and hydrostatic pressures (0.1, 30, 60, and 90 MPa). *P. pentosaceus* XY62 was cultured in MRS medium at 28℃ to logarithmic growth phase (OD 0.5). Then the culture was centrifuged at 4,000 rpm for 5 min, and the supernatant was replaced with the same volume of fresh MRS medium. The culture was transferred to different conditions, cultured for 3 h, and centrifuged to extract RNA. The incubation time was determined based on the growth curve to ensure that the strain was in the logarithmic phase both when placed in and removed from the equipment. It should be emphasized that under hydrostatic pressure conditions, the culture was transferred into a sterile syringe with a polyethylene pipe cap and then placed into the high-pressure vessel (Feiyu Science and Technology Exploitation Co. Ltd., Nantong, China). Total RNA was prepared using the Trizol method. The reverse transcription reaction and RT-PCR were, respectively, conducted using the PrimeScript RT reagent Kit with gDNA Eraser and the TB Green Premix Ex TaqtTM II (Japan TaKaRa BIO, made in China) following the manufacturer’s instructions. The qRT-PCR reactions were performed using a 7500 Fast Real-Time PCR System (Applied Biosystems, Foster City, CA, USA). The 16S rRNA gene was used as the reference gene (44). Oligonucleotide primers were designed using the Primer-BLAST and synthesized by Suzhou jinweizhi Biotechnology Co., Ltd. (Suzhou, China). All measurements were taken using three biological replicates and technical replicates.

### Isolation and characterization of compounds

*P. pentosaceus* XY62 was inoculated into MRS liquid medium until the logarithmic phase. Then, the bacterial broth was transferred into Erlenmeyer flasks containing fresh MRS liquid medium in a 1% ratio (vol/vol) and cultivated at 20°C for 6 days. A total of 10 L of bacterial culture was extracted twice with an equal volume of ethyl acetate and concentrated using a rotary evaporator. The crude extract was segmented using silica gel plates with a combination of solvents, Petroleum Ether/Ethyl Acetate (PET/EtOAc), in various ratios (9:1, 8:2, 7.5:2.5, 7:3, vol/vol). The optimal ratio was determined by the Rf value. Eluents with different ratios were used to fractionate the crude extract (PET/EtOAc = 7.5:2.5, 5.5:4.5, 3.5:6.5, 1.5:8.5, 0:10, vol/vol; EtOAc/MeOH = 8:2, 7:3, 6:4, 4:6, 2:8, 0:10, vol/vol). Ultimately, seven distinct components (1–7) were obtained. Compound 2–1 (190.6 mg, t_R_ = 2.716, MeCN/H_2_O, 0.5:0.95) was purified from component 2 using a 1260 Infinity II HPLC system. The purification was performed using an Agilent Zorbax SB-C18 column (250 × 9.4 mm, 5 µm) at a flow rate of 1 mL/min. UV detection was set at 220 nm, and the temperature was maintained at 40°C. The compound was dissolved in deuterated methanol (CD3OD). Nuclear magnetic resonance (NMR) spectra were obtained using a Bruker 500 spectrometer or a JEOL 600 spectrometer. The ^1^H NMR and ^13^C NMR spectra were analyzed using Mestre Nova software version 12.0. The chemical shifts δ (ppm) are relative to CD3OD (δC 49.00 and δH 3.31).

### Antibacterial assay

Three drug-resistant pathogens, *Salmonella choleraesuis*, *Escherichia coli* MG1655, and *Enterococcus faecalis* FA2-2, along with two typical aquatic enteric pathogens, *Edwardsiella tarda* and *Aeromonas hydrophila*, were used as indicator bacteria. The antibacterial activity of the crude extracts was evaluated using the K-B agar diffusion method. In total, 200 µL of the five indicator bacteria (OD 0.5) were inoculated in LB medium. Then, the sterile circular filter paper was evenly attached to the LB. The crude extract was dissolved in methanol at a concentration of 100 mg/mL, and 6 µL was dripped onto the circular filter paper. After incubating at 37°C for 12 h, the areas of the zone of inhibition were recorded. The antibacterial activity of the monomeric compound was evaluated using the micro-broth dilution method. The compound was dissolved in Dimethyl sulfoxide (DMSO). The positive control was gentamicin, and the negative control was DMSO.

### Drug sensitivity test of *P. pentosaceus*

The antibiotic susceptibility of strain XY62 was tested using the K-B agar diffusion method. Vancomycin, linezolid, ampicillin, tetracycline, gentamicin, lincomycin, chloramphenicol, and erythromycin were selected as the test drugs according to the antibiotic resistance genes of the strains. *P. pentosaceus* XY62 was inoculated into a liquid MRS medium for cultivation, and 200 µL of bacterial solution (OD 0.5) was plated on a solid medium. After the application of sterile circular filter paper, 6 µL of antibiotics (1 mg/mL) and negative controls (dd H_2_O and absolute alcohol) were added on top. The plates were then cultured at 20°C for 24 h. The diameter of the inhibition zone was measured to assess drug resistance.

### Determination of biofilm formation ability of *P. pentosaceus*

The microtiter dish assay was used to determine biofilm formation. *P. pentosaceus* XY62 was cultured in MRS medium to saturation and then diluted 1:50 into fresh medium. In total, 200 µL of cultures were dispensed per well into a 96-well microtiter polystyrene plate, with the MRS medium used as the negative control. The plates were incubated at 28°C for 48 h. The cultures were aspirated and rinsed three times with a 0.85% saline solution to remove unattached cells and assess their ability to form biofilms. Then, 200 µL of a 0.1% crystal violet solution was added to each well and stood for 10 min. After removing the crystal violet, the orifice plate was rinsed with running water. A solution containing 20% methanol and 10% acetic acid was used to dissolve the crystal violet for 15 min after the plate was dried. The absorbance of the plate was measured at 550 nm (45, 46). The ability of *P. pentosaceus* XY62 to form biofilms was determined by the cutoff values. The OD value of the negative control was the optical density cutoff (ODC) (47).

## RESULTS

### Gut microbial community structure of amphipods from the Mariana Trench

An analysis of microbial diversity was conducted to investigate the community structure of gut samples in *H. gigas*. After optimization, 37,026 sequences were obtained from the uncultured microbial samples, with an average sequence length of 428 bp. Taxonomic analysis revealed a limited diversity, with only two phyla, 10 genera, and 12 species identified. Proteobacteria were predominant, accounting for 56.11% of the total, whereas Firmicutes made up the remaining 43.89% (Fig. S1b). The bacterial taxa identified at the genus level in the sample included *Psychrobacter* (56.09%), *Vagococcus* (37.47%), *Macrococcus* (3.93%), *Weissella* (1.17%), *Staphylococcus* (0.39%), *Carnobacterium* (0.32%), *Lactococcus* (0.31%), *Exiguobacterium* (0.28%), *Enterococcus* (0.06%), and *Achromobacter* (0.02%) (Fig. 1b). Furthermore, the culture-dependent assay was performed, and 77 pure isolates were obtained using 23 different media (Fig. 1c; Table S1). Based on 16S rDNA identification and taxonomic classification, there exist three phyla, 13 genera, and 20 species. There were special differences in the bacterial compositions detected by the two approaches. The main phyla identified were Firmicutes (56.63%) and Proteobacteria (42.17%), with Actinomycetes (1.20%) only identified using cultivation techniques (Fig. S1b). At the genus level, the two methods shared seven bacteria genera, including *Psychrobacter*, *Vagococcus*, *Macrococcus*, *Weissella*, *Staphylococcus*, *Lactococcus*, and *Exiguobacterium*. Among these genera, *Psychrobacter* had the highest proportion. The six unique genera of bacteria in cultivation included *Mammaliicoccus*, *Pediococcus*, *Pseudomonas*, *Bacillus*, *Brevibacterium*, and *Citrobacter*, whereas three unique genera of bacteria (*Carnobacterium*, *Enterococcus*, and *Achromobacter*) were only found in microbial diversity analysis (Fig. 1b).

### *P. pentosaceus* XY62 exhibits potential probiotic function

**Fig 2:**
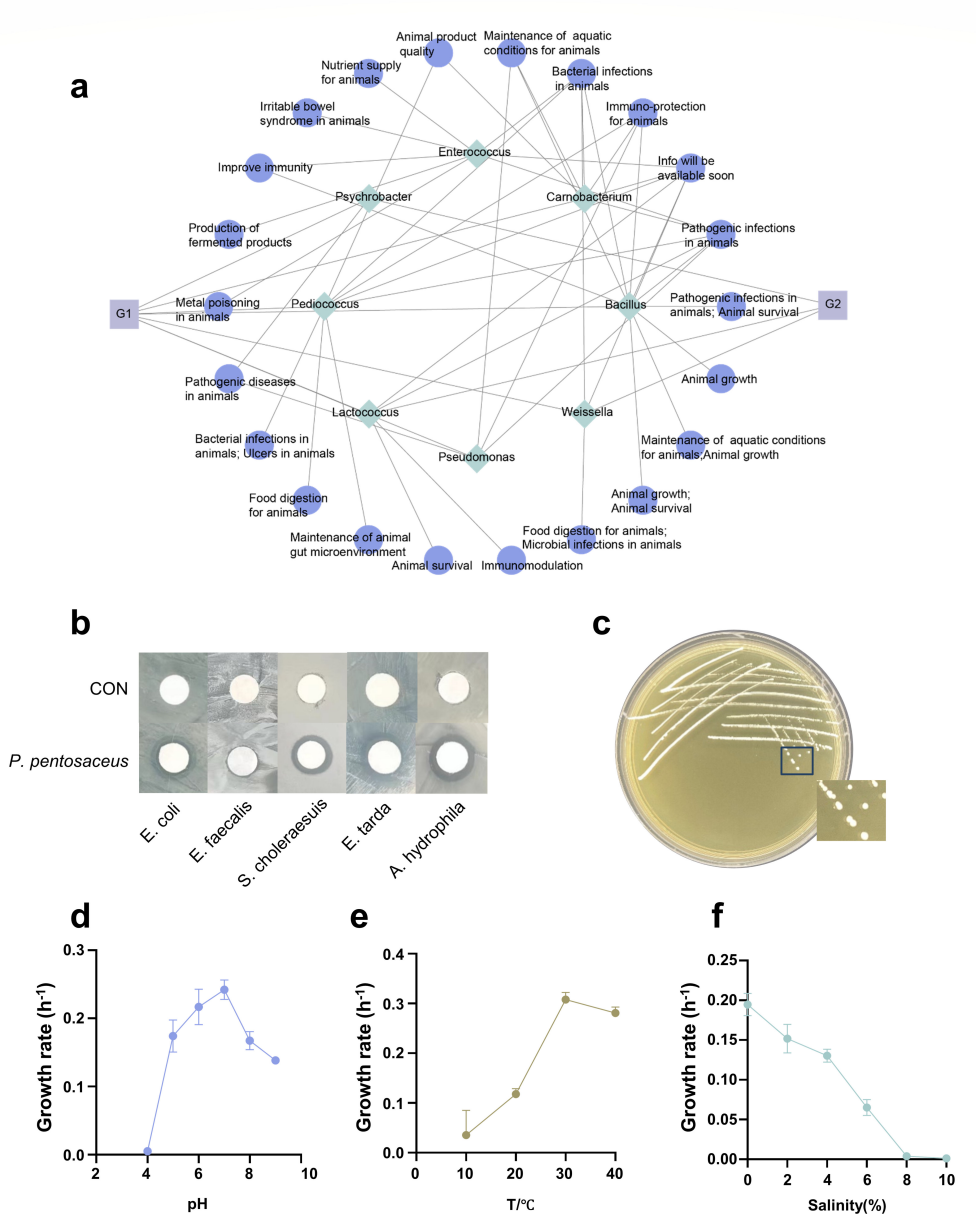
Genetic and physiological characteristics of *P. pentosaceus* XY62. (**a**) Composition and function of probiotics in the gut of hadal amphipods. G1: The microbial culture technology; G2: the 16S rRNA sequencing technology. Cytoscape_v3.8.0. (**b**) The diameter of the inhibition zone of *P. pentosaceus* XY62 against gut pathogens. (**c**) Colony morphology of *P. pentosaceus* XY62 after incubation for 3 days on MRS medium. (**d**) Growth rate of *P. pentosaceus* XY62 at different temperatures. GraphPad Prism 8.0.3. (**e**) Growth rate of *P. pentosaceus* XY62 at different pH values. GraphPad Prism 8.0.3. (**f**) Growth rate of *P. pentosaceus* XY62 at different salinity. GraphPad Prism 8.0.3.

To investigate the ecological function of gut microorganisms in *H. gigas*, a total of eight potential probiotics were identified using the Probio database. Their probiotic functions were mainly associated with “pathogenic diseases,” “bacterial infections,” and “immuno-protection” (Fig. 2a). In addition, these strains also demonstrated the ability to improve food digestion and animal growth. Among them, the genera *Bacillus*, *Pediococcus*, and *Enterococcus* displayed the most abundant probiotic functions, with *P. pentosaceus* being the predominate isolated bacterium (Fig. 1c and 2a).

An antibacterial assay was conducted to evaluate the bioactivities of bacteria isolated from the gut of amphipods. All the isolated bacteria were subjected to a 3-day culture period, after which the fermentation broth was extracted using ethyl acetate. The metabolites of *P. pentosaceus* exhibited inhibitory activity against four indicator bacteria, with the most significant inhibitory activity against *A. hydrophila* (Fig. 2b; Fig. S2a). Compared with *P. pentosaceus*, other gut isolates exhibited minimal antibacterial activity (Fig. S2b). Therefore, *P. pentosaceus* was selected for further research.

Seven strains of *P. pentosaceus* were isolated from five distinct media. The 16S rDNA sequences of these strains had the highest sequence similarity to *P. pentosaceus* DSM 20336, ranging from 99.72% to 99.86% (Table S2). The initially isolated *P. pentosaceus* XY62 was selected for further investigation. After being incubated for 3 days on MRS medium, the colonies appeared circular with a milky white color, opaque appearance, smooth and moist surface, regular edges, and slight convexity and measured 1–2 mm in diameter (Fig. 2c; Fig. S2c). *P· pentosaceus* XY62 could survive within a pH range of 6–9 (Fig. 2d). Specifically, pH 7, 30°C, and 0% salinity were identified as the optimal conditions for growth (Fig. 2e and f; Fig. S3).

### General genetic features and metabolic strategy of *P. pentosaceus* XY62

**Fig 3:**
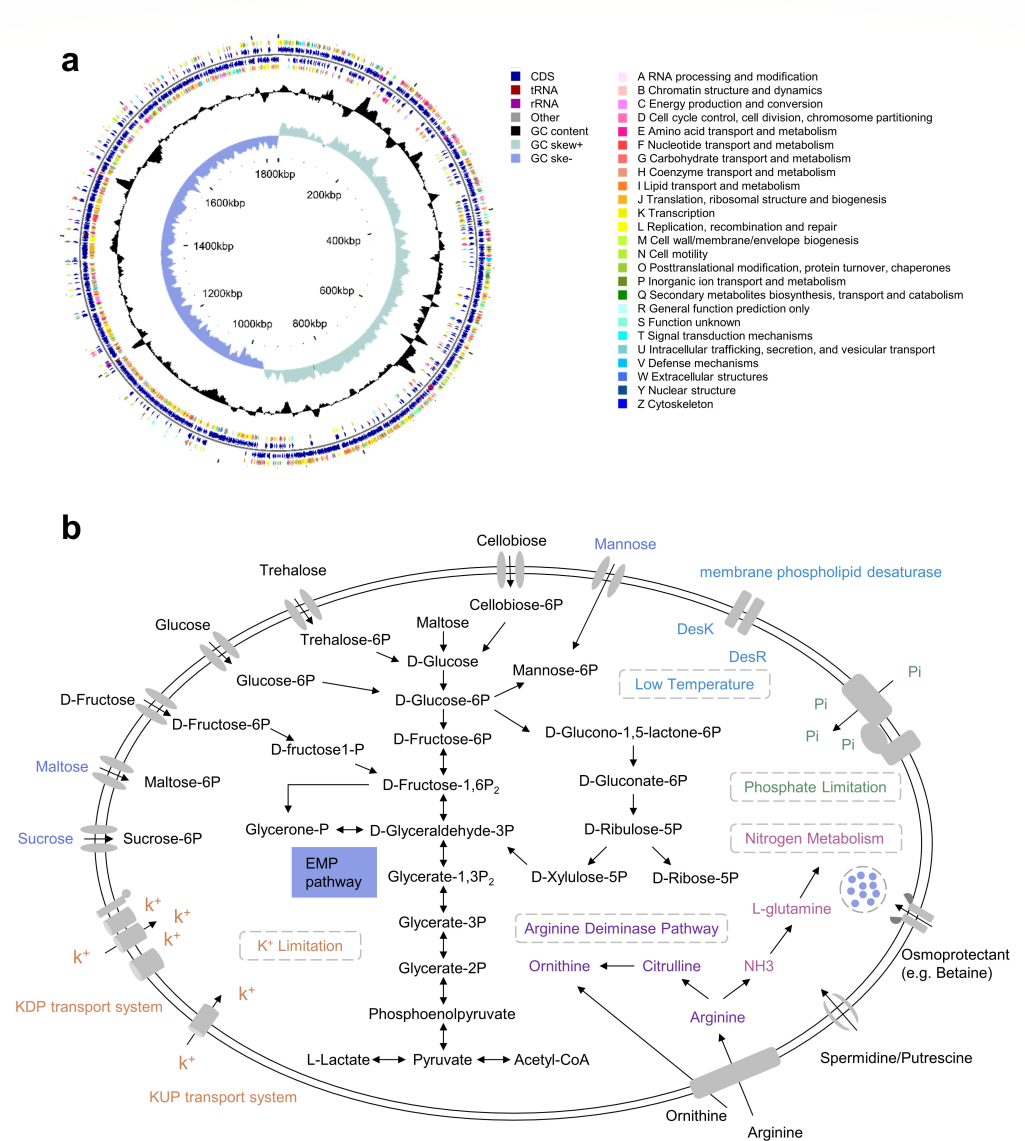
Genome structure and functional pathways of *P. pentosaceus* XY62. (**a**) Chromosomal structure of *P. pentosaceus* XY62. The chromosome size was 1,836,113 bp with a GC content of 37.20% and contained 1,882 genes. A total of 56 tRNA, 15 rRNA, and 32 sRNA were predicted in the genome. 10 tandem repeats, with a total length of 2,185 bp, were detected, accounting for 0.13% of the genome. cloud.majorbio.com. (**b**) The diagram of the environmental adaptation strategy of *P. pentosaceus* XY62 based on the genome.

The genome of *P. pentosaceus* XY62 was characterized through whole-genome sequencing and assembly. The genome sequence of *P. pentosaceus* XY62 was assembled into one chromosome and two plasmids. The size of the chromosome was 1,836,113 bp with a GC content of 37.20% (Fig. 3a). Plasmid A was identified as a novel plasmid with a length of 53,759 bp and a GC content of 39.16% (Fig. S4a). The length of plasmid B was 9,046 bp and the GC content was 34.60% (Fig. S4b). A total of 1,882 genes were identified in *P· pentosaceus* XY62, with an overall length of 1,665,843 bp. According to the EggNOG and KEGG results, most genes of *P. pentosaceus* XY62 are associated with metabolism, information storage and processing, and cellular processes and signaling (Fig. S4c and d).

Given the complex trench environment, we inferred that *P. pentosaceus* XY62 has the corresponding metabolic capacity and environmental adaptation strategies. The metabolic pathway showed a wide range of carbon source utilization, including glucose, trehalose, and cellobiose. Although *P. pentosaceus* XY62 cannot utilize sucrose, maltose, and mannose as carbon sources, it can be transported through the phosphotransferase system, which may be used to maintain cellular osmotic regulation. A total of 39 genes belonging to three CAZyme superfamilies have been identified. Among these, three genes were identified as members of the Carbohydrate Esterases (CEs) family, whereas 18 genes were assigned to the Glycoside Hydrolases (GHs) family, and another 18 genes belonged to the Glycosyl Transferases (GT) family (Fig. S5a).

The osmoprotectant ABC transport system (opuA, opuC, and opuBD) and a BCCT family betaine/carnitine transporter were identified from its genome, which controls the input of osmoprotectant from the environment. In addition, the KUP/HAK/KT family potassium transporter (KUP) and the KDP potassium transport system (kdpABC/kdpDE) can maintain osmotic balance by transporting potassium ions. The two-component system DesK-DesR (Nar L family) regulates the synthesis of unsaturated fatty acids. It can respond to low-temperature stress, which is essential for the survival of *P. pentosaceus* XY62. Complete spermidine/putrescine transporters including permidine/putrescine-binding periplasmic protein, permease protein, and ATP-binding protein were annotated in the genome. This suggests that *P. pentosaceus* XY62 can regulate osmotic pressure and signal transduction by transporting polyamines. The *arcDABC* operon, which is involved in the transport of arginine and ornithine, supplies energy for *P. pentosaceus* XY62 under anaerobic conditions. Ammonia produced in this process is an important compound that links to other metabolic pathways. A high-affinity Pi transport system (PstS system) and the PhoB/PhoR two-component regulators in the genome ensure the absorption of phosphorus under phosphorus limiting conditions (Fig. 3b). Based on the RAST database, 31 genes linked to stress response were annotated. Among these, 25.81% were associated with osmotic stress, 32.36% with oxidative stress, 3.22% with cold shock, 35.48% with heat shock, and 3.22% with detoxification genes (Fig. S5b).

### Comparative genomics reveals unique environmental adaptation mechanisms

**Fig 4:**
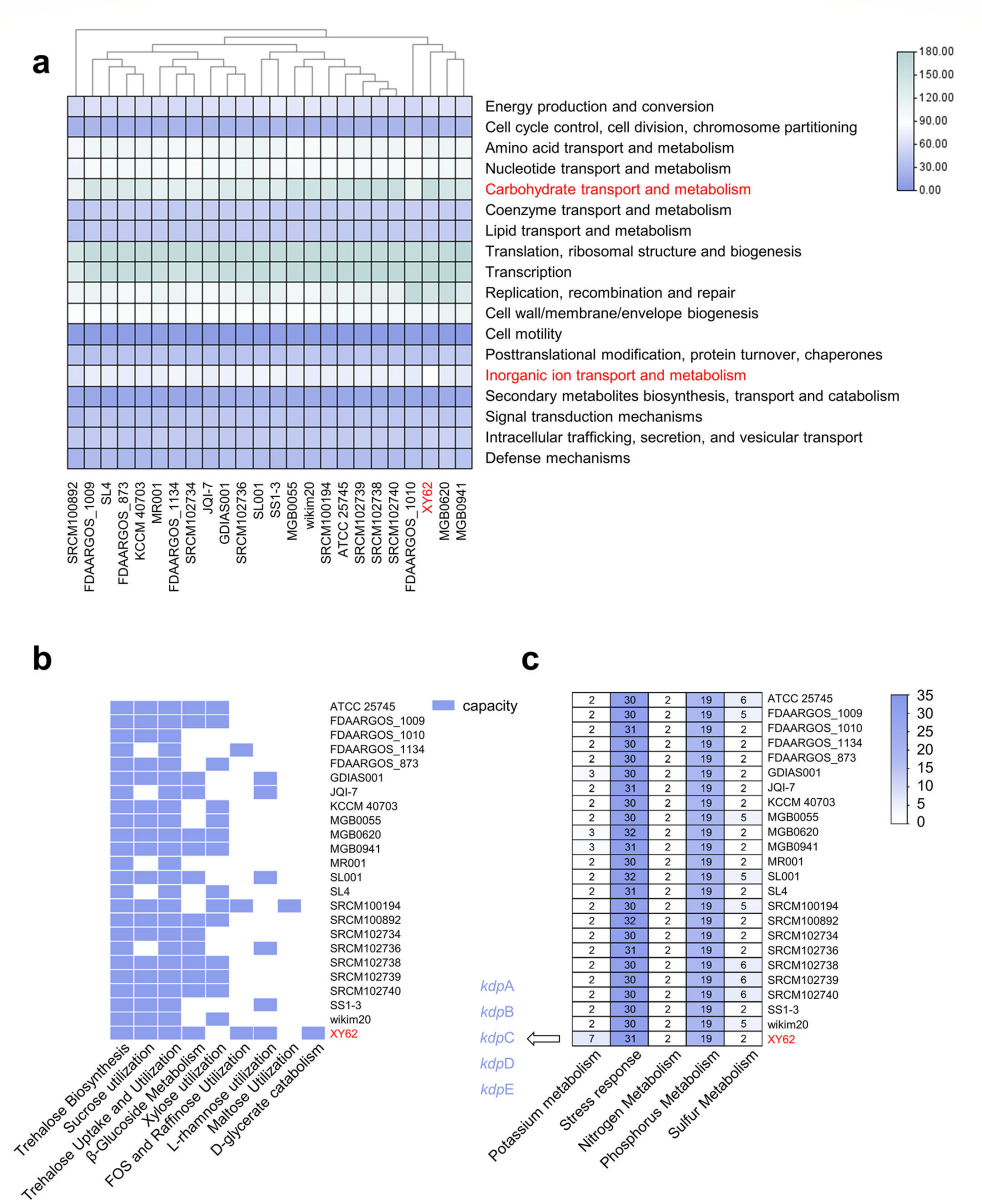
Comparative genomic analysis of gene function in 24 *P. pentosaceus* strains. (**a**) Heat map of COG secondary functional differences among 24 strains. Tbtools. (**b**) Oligosaccharide metabolism capability of 24 strains. TBtools. (**c**) Genes related to environmental stress and element metabolism in 24 strains. *kdp*A, *kdp*B, *kdp*C, *kdp*D, and *kdp*E are unique genes of strain XY62, which constitute a complete KDP potassium ion transport system. TBtools.

To further explore the environmental adaptation characteristics of *P. pentosaceus* XY62, we compared its whole genome with 23 strains of *P. pentosaceus* from diverse origins in the NCBI database (Table S3). The gene functions of 24 strains were compared using the eggNOG and the KEGG database. Except for those with unannotated genes, all strains shared a total of 763 COG genes and 643 KEGG genes. *P. pentosaceus* XY62 was annotated with 1,036 COG genes and 910 KEGG genes, possessing the highest number of function genes in comparison to other strains (Fig. S6a and b). Based on COG annotations, the genes of all strains were highly enriched in “translation, ribosomal structure, and biogenesis,” “transcription," and “carbohydrate transport and metabolism." In contrast to other strains, *P. pentosaceus* XY62 displayed a higher gene enrichment in “carbohydrate transport and metabolism” and “inorganic ion transport and metabolism,” which may be an advantage for its adaptation to deep-sea environments (Fig. 4a). At the first hierarchical level of the KEGG pathway, all strains had abundant genes in “metabolism,” “genetic information processing,” and “environmental information processing.” At the second hierarchical level of the KEGG pathway, *P. pentosaceus* demonstrated a higher abundance of genes associated with carbohydrate metabolism (Fig. S6c).

A total of 39 CAZyme families were identified across 24 strains, with 12 families being present in all strains. Among them, the GT2 and the GT4 families were the two largest families. Interestingly, the CE2 (acetyl xylan esterase) family was present in all strains except *P. pentosaceus* XY62, whereas the GH78 (α-L-rhamnosidase) family was exclusively found in *P. pentosaceus* XY62 (Fig. S7). The oligosaccharide and monosaccharide metabolism of 24 strains were analyzed based on the RAST database. All 24 strains exhibited the capacity to utilize and synthesize trehalose. Strain XY62 was unable to utilize xylose and maltose as carbon sources but was able to consume the widest variety of carbon sources (Fig. 4b). Its extensive carbon source degradation profile may be crucial for survival in the Hadal Trench. Furthermore, only strain XY62 exhibited a distinct capability to metabolize D-Glycerate, attributed to the presence of the *hpr*A (glycerate dehydrogenase) in its genome (Fig. S8). Additionally, *P. pentosaceus* possessed numerous genes involved in stress response and phosphorus metabolism. When comparing potassium metabolism genes, it was found that strain XY62 had a variety of potassium transport systems, and *kdp*A, *kdp*B, *kdp*C, *kdp*D, and *kdp*E were unique to *P. pentosaceus* XY62 (Fig. 4c).

### The expression of stress resistance genes revealed the environmental adaptability of *P. pentosaceus* XY62

Bacteria encounter a variety of stressors within their complex ecological niches, requiring the deployment of multiple defense mechanisms to overcome environmental challenges. The specific roles of the predicted environmental response genes of *P. pentosaceus* XY62 under various environmental pressures remain incompletely understood. The environmental response genes including *opu*A (osmoprotectant transport system ATP-binding protein), *msr*B (response to oxidative stress), gene *1886* (peroxide stress protein), *dan*K (molecular chaperone DnaK), and *csp*A (cold shock protein), as well as the unique genes *hpr*A (glycerate dehydrogenase) and *kdp*A (potassium-transporting ATPase subunit), were selected to examine the strain’s response to environmental changes.

**Fig 5:**
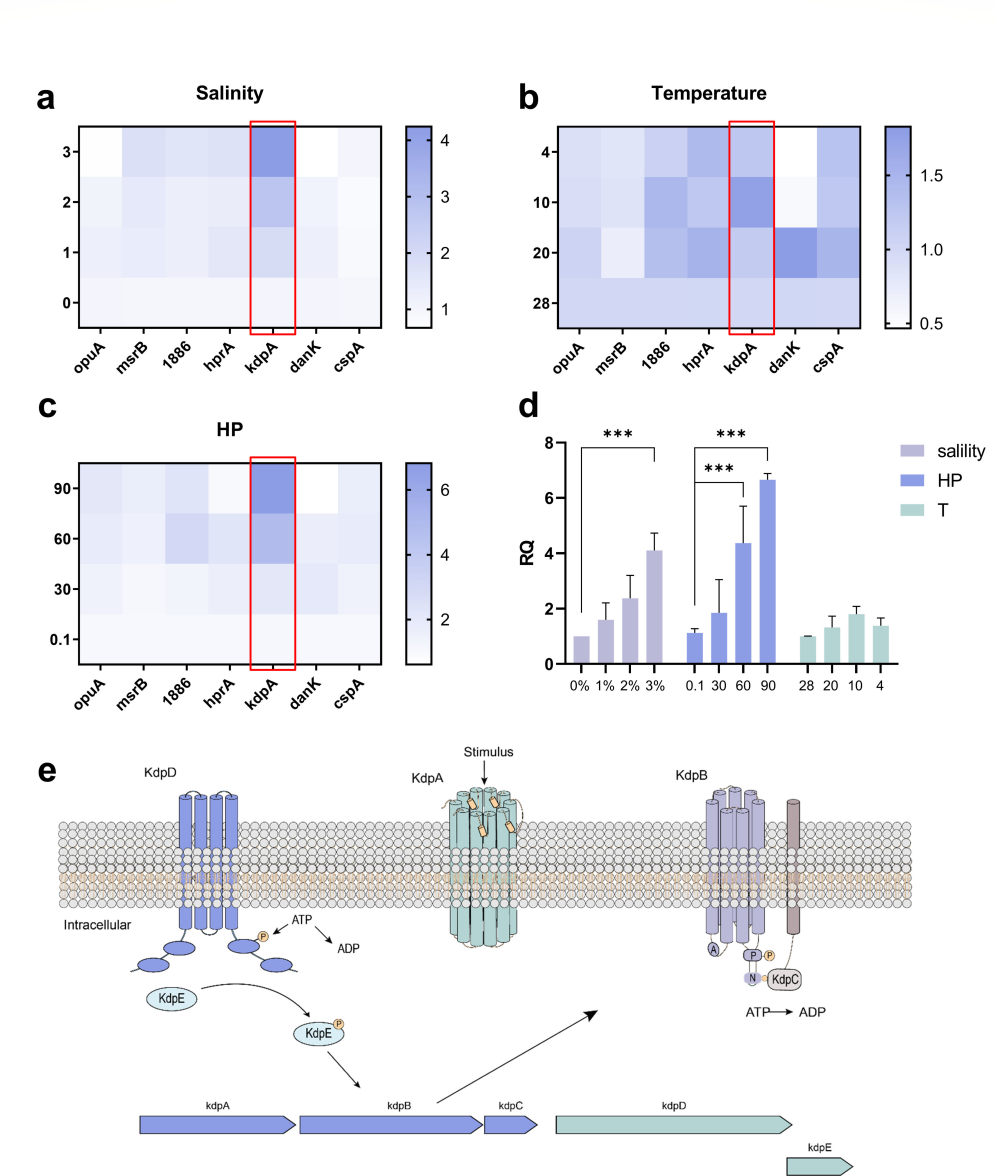
Expression of stress resistance genes in *P. pentosaceus* XY62 under various environmental stressors. (**a**) Expression of stress resistance genes in *P. pentosaceus* XY62 under salinity stress. GraphPad Prism 8.0.3. (**b**) Expression of stress resistance genes in *P. pentosaceus* XY62 under temperature stress. GraphPad Prism 8.0.3. (**c**) Expression of stress resistance genes in *P. pentosaceus* XY62 under hydrostatic pressure stress. GraphPad Prism 8.0.3. (**d**) Gene expression of the *kdp*A gene under varying levels of salinity, temperature, and hydrostatic pressure. GraphPad Prism 8.0.3. (**e**) Structure of the KDP potassium ion transport system in *P. pentosaceus* XY62. KdpA is mainly responsible for the transport of K + ions; KdpB is a P-type ATPase that binds to and hydrolyzes ATP; KdpC is anchored to the membrane, which can increase the affinity of KdpB for ATP; KdpD is a histidine kinase that gets phosphorylated upon external stimuli and then transfers the phosphate group to the response regulator KdpE. Modified in Adobe Illustrator 2021.

Upon increasing the salinity to 3%, the expression level of *mrs*B elevated by 1.7-fold, gene *1886* by 1.6-fold, *hpr*A by 1.7-fold, and *kdp*A by a significant 4.1-fold, highlighting their roles in adapting to changes in salt concentrations (Fig. 5a). Similarly, with a decrease in culture temperature from 28°C to 4°C, the expression levels of gene *1886*, *hpr*A, *kdp*A, and *csp*A all increased by 1.3-fold (Fig. 5b). In comparison to 0.1 MPa, under 90 MPa, *kdp*A was notably upregulated by 5.9-fold, *csp*A by 1.7-fold, *opu*A by 1.8-fold, *mrs*B by 1.5-fold, and gene *1886* by 2.0-fold, demonstrating their diverse roles in response to environmental stressors (Fig. 5c). In particular, the genes *kdp*A and *gene 1886* exhibited simultaneous responses to variations in salinity, temperature, and hydrostatic pressure. Among these genes, the expression level of *kdp*A showed the most significant variations in response to different salinity conditions (0/2%, *P* < 0.001; 0/3%, *P* < 0.001) and pressure conditions (0.1/60 MPa, *P* < 0.001; 0.1/90 MPa, *P* < 0.001) (Fig. 5d). These results indicate the strong impact of potassium-transporting ATPase (KDPA) on the response of *P. pentosaceus* XY62 to the extreme environment. KDPA belongs to the KDP potassium ion transport system and is primarily responsible for transporting potassium ions. According to the genome annotation and previous studies (48), we conducted the KDP potassium ion transport system of *P. pentosaceus* XY62 (Fig. 5e).

### *P. pentosaceus* XY62 shows antibacterial activities

**Fig 6:**
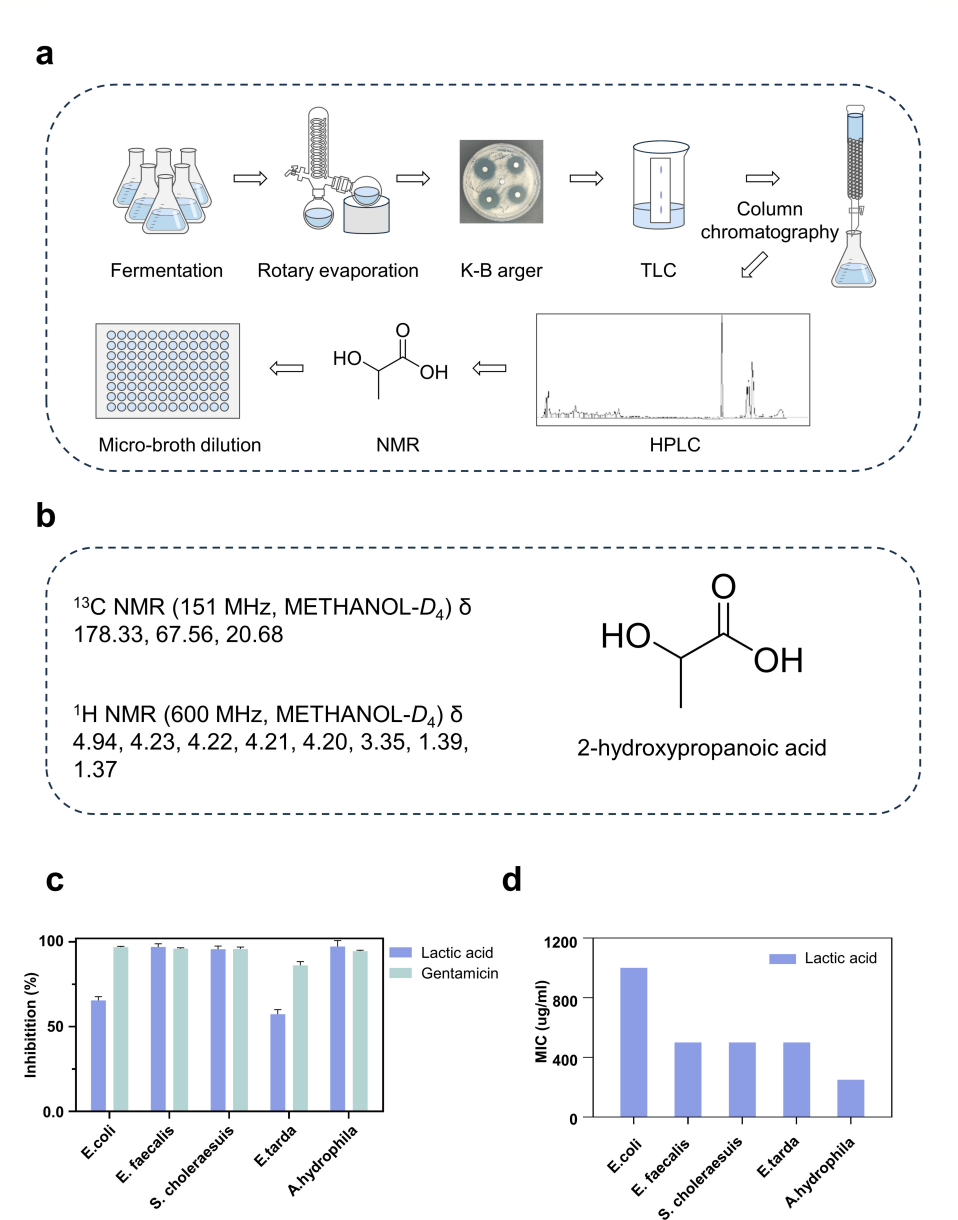
Isolation and purification of metabolites from *P. pentosaceus* XY62 and antibacterial activity of compounds. (**a**) Workflow chart of metabolite separation. (**b**) The chemical shifts of carbon and hydrogen spectra and molecular structure of the isolated compound. ChemDraw 19.0. (**c**) The antibacterial activity of lactic acid at a concentration of 500 µg/mL. GraphPad Prism 8.0.3. (**d**) The half-maximal inhibitory concentration of lactic acid. GraphPad Prism 8.0.3.

To investigate the antibacterial characteristics of *P. pentosaceus* XY62, the fermentation broth was extracted and subsequently fractionated into seven components using a silica gel column sourced from 12.243 g of crude extract (Fig. 6a). Components 2 (6–22) and 3 (15, 23–51) exhibited inhibitory effects on the indicator bacteria (Fig. S9). Compound 1 was characterized as a clear liquid with UV absorption at 220 nm and a molecular formula of C_3_H_6_O_3_ (90.078) (Fig. S10). The NMR assay was carried out. Its 1H NMR spectrum (600 MHz, METHANOL-D4) displayed peaks at δ 4.94, 4.23, 4.22, 4.21, 4.20, 3.35, 1.39, and 1.37 (Fig. 5b; Fig. S11a). The 13C NMR (151 MHz, METHANOL-D4) displayed peaks at δ 178.33, 67.56, and 20.68 (Fig. 5b; Fig. S11b). Based on the chemical characterization and comparison with the existing data, compound 1 was identified as lactic acid (Fig. 6b).

The microbroth dilution method was used to evaluate the antibacterial efficacy of lactic acid. The inhibitory rates of lactic acid on various bacteria were as follows: *E. faecalis* (97%), *S. choleraesuis* (95%), and *A. hydrophila* (97%) at a concentration of 500 µg/mL (Fig. 6c). The inhibitory rates for *E. coli* and *E. tarda* were all over 50%. Notably, lactic acid showed the most potent inhibitory effect on the indicator bacteria *A. hydrophila*, with a minimum inhibitory concentration (MIC) of 250 µg/mL (Fig. 6d). Furthermore, the biosynthetic gene cluster for type III polyketide synthases (T3PKS) and the Enterolysin A (En1A) type bacteriocin were predicted in the genome of XY62 (Fig. S12a and b). T3PKS and En1A have been found to participate in the biosynthesis of antibacterial agents or bacteriocin, providing evidence for XY62’s ecological function as a defense mechanism and potentially even promoting the health of the host.

### The self-defense mechanism during the colonization process

**Fig 7:**
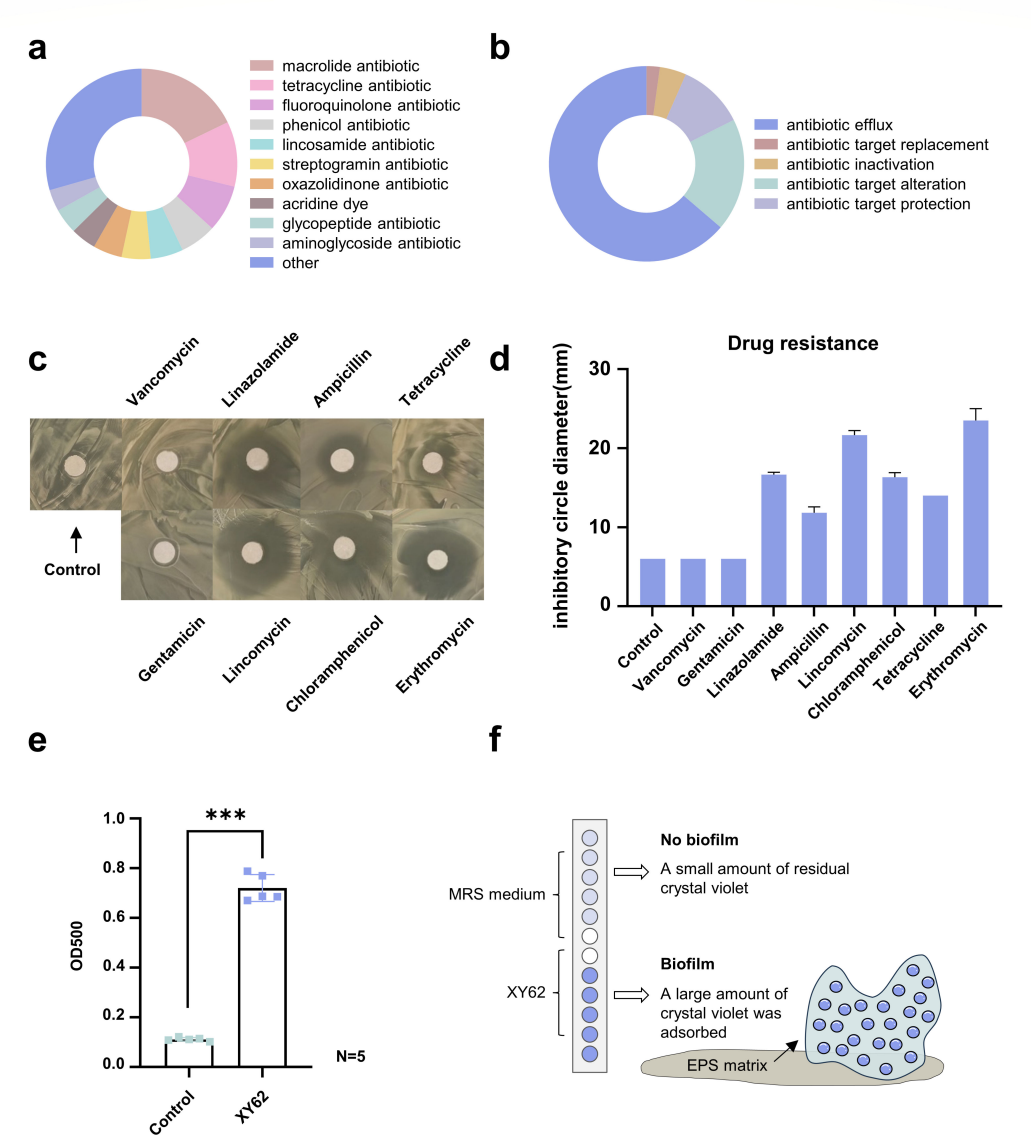
Drug resistance and biofilm formation ability of *P. pentosaceus* XY62. (**a**) The types of resistance genes of *P. pentosaceus* XY62. GraphPad Prism 8.0.3. (**b**) The resistance mechanisms of *P. pentosaceus* XY62. GraphPad Prism 8.0.3. (**c**) The inhibitory effect of antibiotics against *P. pentosaceus* XY62. (**d**) The diameter of the inhibition zone of antibiotics against *P. pentosaceus* XY62. GraphPad Prism 8.0.3. (**e**) Biofilm formation ability of *P. pentosaceus* XY62. Based on the range of optical density, it is possible to identify whether *P. pentosaceus* XY62 produces non-biofilm (OD ≤ ODC), weak biofilm (ODCOD ≤ 2 < ODC), moderate biofilm (2ODCOD ≤ 4 < ODC), or strong biofilm (4ODCOD). GraphPad Prism 8.0.3. (**f**) The biofilm of *P. pentosaceus* XY62.

Recent studies have shown that antibiotic resistance genes (ARGs) are highly diverse in the bottom seawater (below 9,600 m) of the Mariana Trench (49). Microorganisms in the Hadal Zone, displaying antibiotic resistance, could potentially enhance their capacity to survive. Here, a total of 90 ARGs were predicted in the genome of *P. pentosaceus* XY62. These genes are associated with resistance to different drugs, including macrolide antibiotics (32.22%), tetracycline antibiotics (20.00%), fluoroquinolone antibiotics (14.44%), phenicol antibiotics (11.11%), lincosamide antibiotics (10.00%), etc. (Fig. 7a). Additionally, the predominant resistance mechanisms in XY62 included antibiotic efflux (64.44%), antibiotic target alteration (18.89%), antibiotic target protection (11.11%), antibiotic inactivation (4.44%), and antibiotic target replacement (2.22%) (Fig. 7b). Eight clinically relevant antibiotics were selected for a tolerance test. *P. pentosaceus* was able to grow in 1 mg/mL of vancomycin, gentamicin, and tetracycline, indicating some tolerance to glycopeptide antibiotics, aminoglycoside antibiotics, and tetracycline antibiotics (Fig. 7c and d).

Microbial biofilms are communities of aggregated microbial cells. Genes involved in biofilm formation and regulation were annotated in the genome of *P. pentosaceus* XY62 (gene *0042*, gene *0298*, gene *0314*, and gene *0715*). Based on the results of the microtiter dish assay, the OD value of XY62 (OD550≈0.72) was approximately seven times higher than that of the negative control (ODC500≈0.11) after 48 h of culture at 28°C (*N* = 5; *P* value < 0.001). The negative control was MRS medium, only a small amount of crystal violet remained, whereas a large amount of crystal violet was recruited by strain XY62. It indicates that *P. pentosaceus* XY62 exhibited a remarkable capacity for biofilm production (Fig. 7e and f). We speculate that the biofilm produced by *P. pentosaceus* XY62 can protect gut microorganisms from various extreme environmental stressors.

## DISCUSSION

The Hadal Zone is characterized by low temperatures, high hydrostatic pressure, high salinity, and oligotrophy (5). Amphipoda is widely distributed throughout the trenches and has adapted effectively to its distinctive ecological niche, making it a focal point for studying environmental adaptation in the Hadal Zone (50–52). The gut microbiota plays important roles in maintaining the host’s nutrition, health, and metabolism, as well as in supporting ecological adaptation. However, there is limited research into the functional role of gut microbes in Amphipoda. In this study, we used 16S rRNA sequencing technology and culturing techniques to examine the composition of the gut microflora in Amphipoda. By combining the two methods, the microbial species composition of the amphipods gut was identified, and many pure, culturable strains were obtained. This will enable further research on their ecological function and adaptability.

The 16S rRNA sequencing technology plays an important role in revealing the gut microbial structure of amphipods in the Hadal Trench. However, pure isolated strains can provide deeper insights into microorganisms’ functional and ecological roles (53). In this study, one of the isolated gut strains, *P. pentosaceus,* was characterized with the highest antimicrobial activity. Although *Pediococcus* sp. was identified in the gut of *H. gigas* in the Mariana Trench in 2021, its probiotic effects and capacity to adapt environmentally to deep-sea Amphipoda remain unexplored (29). The genome analysis and purification of the metabolic products of *P. pentosaceus* showed that it could inhibit gut pathogens by producing a variety of active substances, including lactic acid and Enterolysin A. Genome-wide and comparative genomic analyses are increasingly being used in the study of bacterial ecological adaptation to extreme environments (54–56). The research on *Pediococcus* sp. adaptability has primarily focused on environments such as beer and cheese (57, 58). However, its adaptation mechanism in the trench niche has not been studied. The genome of *P. pentosaceus* XY62 contains stress genes that facilitate its adaptation to the extreme environment of the Hadal Zone, including osmotic, cold, and oxidative stress. The arginine deiminase pathway enables *P. pentosaceus* XY62 to obtain energy under anaerobic conditions, whereas the high-affinity phosphorus transport system ensures its phosphorus supply.

Compared with *P. pentosaceus* from other sources, strain XY62’s genome contains a higher number of genes related to carbohydrate transport and metabolism, especially in oligosaccharide metabolism. This suggests that the strain has a greater potential to obtain energy from a wider range of carbon sources, enabling it to thrive in unusual nutritional environments. In addition, the plasmid of *P. pentosaceus* XY62 contains a KDP system, which is a high-affinity transport system for K + ions. Previous studies have confirmed the KDP system’s response to high osmotic stress (59, 60). In 2021, de Araujo et al. highlighted the upregulation of the *kdp* operon expression under low temperatures (61). In our study, we found that *kdp*A not only responds to low temperature and hyperosmotic stress but also, notably, reacts to changes in hydrostatic pressure. The expression of *kdp*A increases under higher hydrostatic pressure, indicating that the KDP system may help bacteria adapt to high-pressure environments.

The Hadal Zone features complex environmental characteristics, subjecting organisms to multiple environmental pressures simultaneously (62, 63). Understanding how microorganisms respond to complex environments is crucial for comprehending their ecological significance and environmental adaptability (64, 65). *P. pentosaceus XY62* demonstrates a range of environmental adaptation mechanisms. Our research revealed that it can activate genes linked to osmotic stress, oxidative stress, and low-temperature stress when faced with high hydrostatic pressure. Studies have indicated that high hydrostatic pressure and low temperature can increase intracellular free radicals, leading to oxidative stress (66, 67). We observed that gene *1886* (oxidative stress protein) can respond simultaneously to high osmotic pressure, low temperature, and high hydrostatic pressure. This suggests that the antioxidant mechanism may play an important role in bacterial adaptation to the Hadal Zone. The gene *hpr*A is involved in energy and substance metabolism in organisms (68). Limited research has been conducted on its environmental response. The specific existence of this gene in strain XY62 and its upregulation under low temperature and high osmotic pressure indicate its potential involvement in environmental stress resistance.

Antibiotic-resistant genes are widespread in the microbiome of the Hadal trench (69). The antibiotic resistance genes and multiple resistance mechanisms in the genome of *P. pentosaceus* may facilitate the bacterium to adapt to challenging environments and compete within the gut niche, but the mechanisms need further investigation. Significantly, the colonization of gut microorganisms is crucial for their growth, development, and probiotic effect (70). Polystyrene microtiter plate assay confirmed the high biofilm production capacity of *P. pentosaceus*. Biofilms can not only assist probiotics in competing for attachment sites but also play roles in resisting extreme environments such as low temperatures, oligotrophic conditions, and high pressure (71, 72). We hypothesize that the formation of biofilms by *P. pentosaceus* XY62 is beneficial for maintaining the gut homeostasis of Amphipoda and improving the adaptation of gut microbiota to abyssal environments. However, the molecular mechanism of biofilm regulation and its role in Hadal environmental adaptation still needs to be explored.

In conclusion, our work has improved the understanding of the structure and function of gut microbiota in the Amphipoda of the trenches and has advanced research on the environmental adaptation mechanism of gut probiotics in the Hadal environment. Notably, we used 16S rRNA technology and the culture-dependent method to investigate the gut microflora of Amphipoda in the trench simultaneously, which effectively revealed the microbial diversity. This approach addresses the limitations of using a single method and provides a more precise understanding of the microflora structure. The results of whole-genome sequencing and comparative genomics revealed the probiotic effect and environmental adaptability of *P. pentosaceus* XY62. In addition, we have demonstrated, the responsiveness of the KPD system to high hydrostatic pressure for the first time, which may aid bacteria in adapting to extreme-depth environments. The gene expression changes indicated that *P. pentosaceus* XY62 can adapt to complex environmental changes by activating a range of stress resistance mechanisms, which may be crucial for its survival in abyssal environments. Moreover, the separation and characterization of metabolites, along with qRT-PCR, confirmed the antibacterial activity and ecological roles of *P. pentosaceus* XY62. In summary, our research has advanced the understanding of the ecological functions and adaptation mechanisms of gut microbiota in hadal amphipods, promoting the optimized utilization of deep-sea resources and the development of bacterial products.

## Data Availability

Unless specifically stated, each experiment was independently repeated three or more times, and data were collected from three biological replicates and three technical replicates. All sequencing data related to the project were stored in the National Center for Biotechnology Information (NCBI) sequence reading archive database. The flora diversity data item number is PRJNA1082922, and the genome data item number is PRJNA1083022. The genome has been assigned the following GenBank accession numbers: Chromosome (CP146862), plasmidA (CP146863), and plasmidB (CP146864).

## SUPPLEMENTAL MATERIAL

Supplemental figures and tables spectrum.00723-24-s0001.docx Fig. S1 to S12; Tables S1 to S4.

ASM does not own the copyrights to Supplemental Material that may be linked to, or accessed through, an article. The authors have granted ASM a non-exclusive, world-wide license to publish the Supplemental Material files. Please contact the corresponding author directly for reuse.
