# Supplementary material for: Microbial community structure and functional traits involved in the adaptation of culturable bacteria within the gut of amphipods from the deepest ocean
Source: Microbiol Spectr. 2024 Dec 10;13(1):e00723-24. doi: 10.1128/spectrum.00723-24 (PMC11705852; doi:10.1128/spectrum.00723-24)
Supplement: Supplemental figures and tables — Fig. S1 to S12; Tables S1 to S4. [file spectrum.00723-24-s0001.docx]

**Microbial community structure and functional traits involved in the adaptation of culturable bacteria within the gut of amphipods from the deepest ocean**

Yukun Cui, Yu Xiao, Zhuo Wang, Paiyao Ji, Changhao Zhang, Yongqi Li, Jiasong Fang, and Xi Yu^*^

**running head:** Adaptability of gut bacteria in hadal amphipods

**Address**: ^1^Shanghai Engineering Research Center of Hadal Science and Technology, College of Oceanography and Ecological Science, Shanghai Ocean University, Shanghai, 201306, China

*Corresponding author: XY (x[yu@shou.edu.cn](mailto:yu@shou.edu.cn), +8615332036650);

**Key words:** gut microbes, amphipods, Mariana Trench, genome, environmental adaptation

**
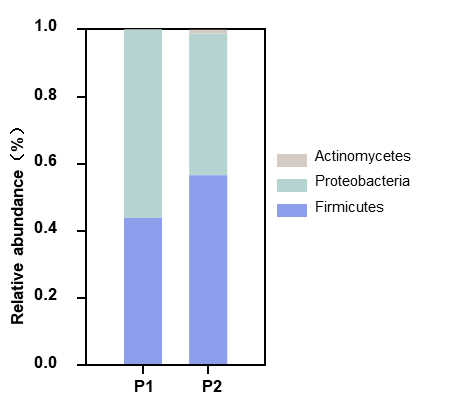
**
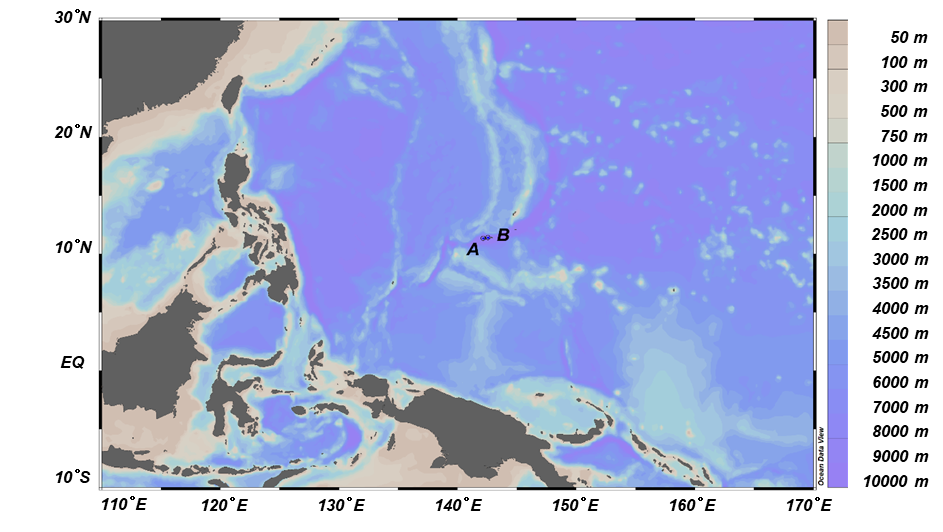
**Fig. S1**

**a**

**b**

**Supplementary Figure 1** Sample site information and gut microbial composition at the phylum level (a) Sampling sites of *H.gigas* from the Mariana Trench (A: 10895m, 142°11.283′E, 11°19.6098′N; B: 10901m, 142°35.3102′E, 11°22.584′N). ODV 5.6.7. (b) Gut bacterial community structure detected by 16S rRNA sequencing technology and culturing techniques (P1: Phylum levels determined by 16S rRNA sequencing technology; P2: Phylum levels determined by microbial culture technology). GraphPad Prism 8.0.3.


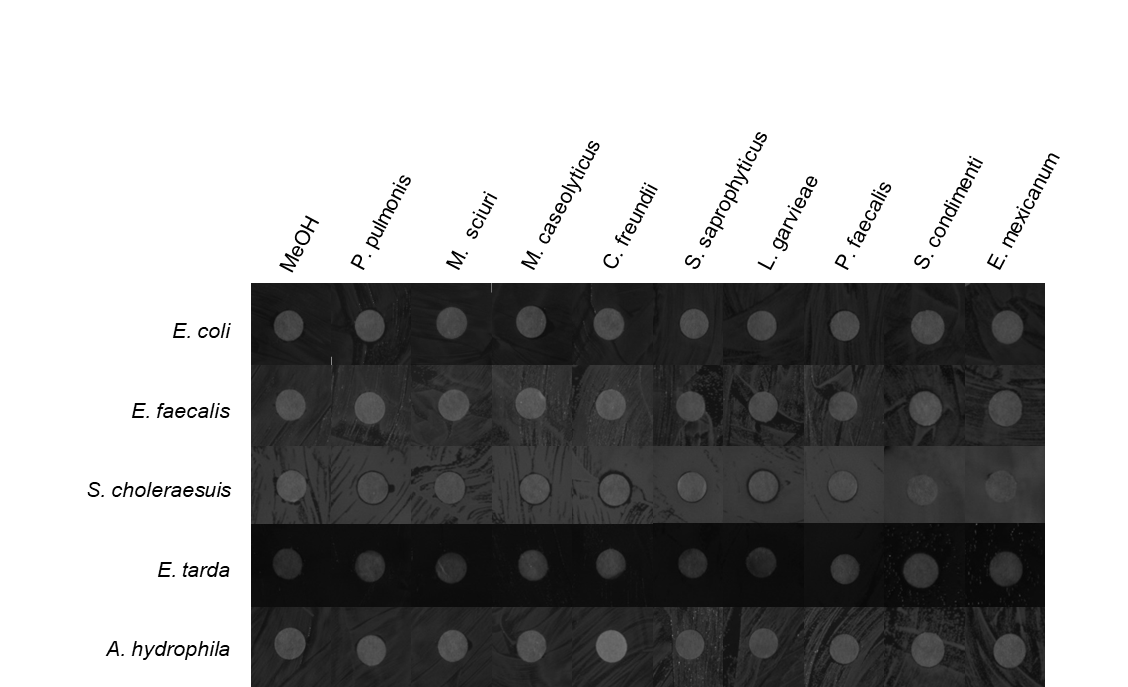

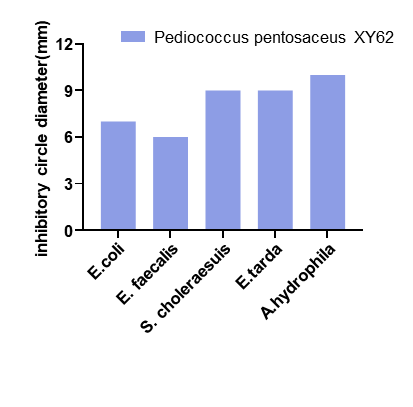
**Fig. S2**

**b**

**c**

**a**


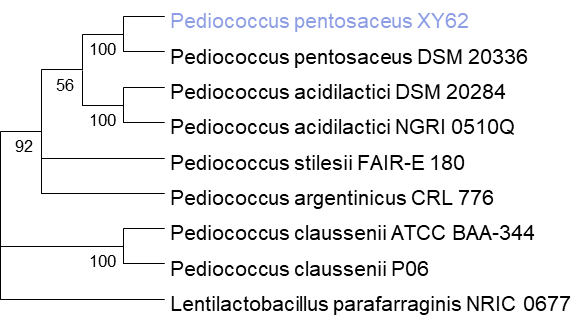


**Supplementary Figure 2** The probiotic activity of gut bacteria in amphipoda. (a) The inhibitory effect of metabolites produced by *P. pentosaceus* XY62. GraphPad Prism 8.0.3. (b) The antibacterial effect of gut bacteria except *P· pentosaceus*. (c) Neighbor-joining tree based on 16S rDNA gene sequences of *P. pentosaceus* XY62. *Lentilactobacillus parafarraginis* NRIC 0677 was used as the outgroup. MEGA11.

**Fig. S3**

**a**

**c**

**b**

**a**


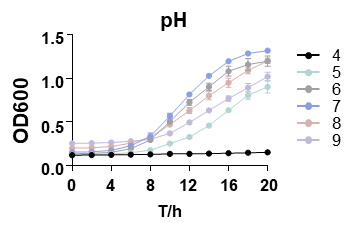


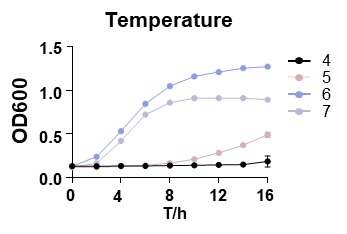


**b**


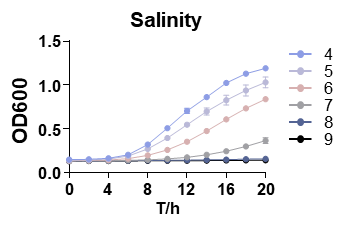


**c**

**Supplementary Figure 3** The optimal growth conditions of *P. pentosaceus* XY62. (a) Growth curves under different pH values. GraphPad Prism 8.0.3. (b) Growth curves under different temperature. GraphPad Prism 8.0.3. (c) Growth curves under different salinity. GraphPad Prism 8.0.3.


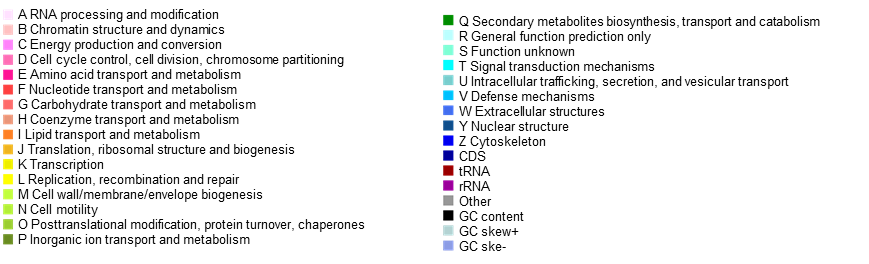

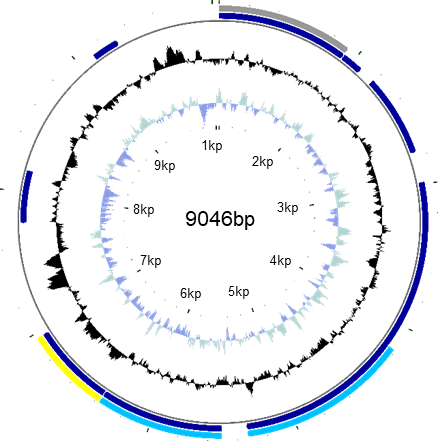

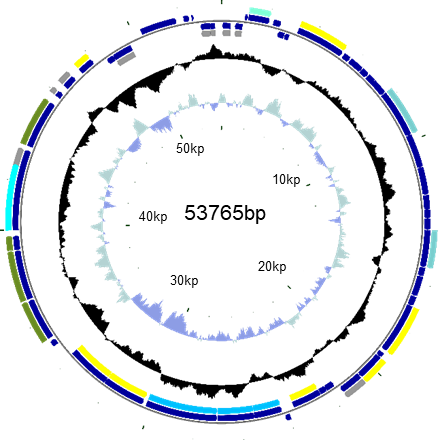
**Fig. S4**

**b**

**a**


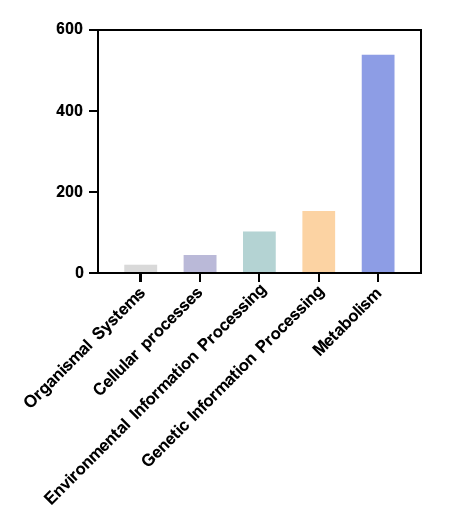

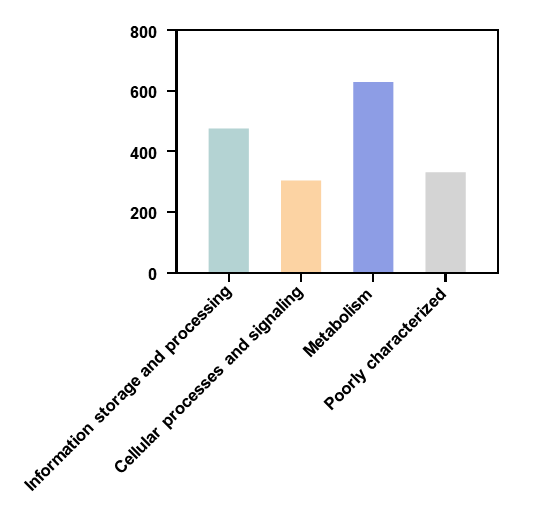


**d**

**c**

**Supplementary Figure 4** Classification of plasmid structures and functional genes in *P. pentosaceus* XY62. (a) Plasmid A has a length of 53,759 bp and a GC content of 39.16%. cloud.majorbio.com. (b) Plasmid B has a length of 9,046 bp and a GC content of 34.60%. cloud.majorbio.com. (c) Classification of COG functions of *P. pentosaceus* XY62. A total of 1586 genes were annotated, 476 genes (30.01%) were involved in information storage and processing, 305 genes (19.23%) were involved in cellular processes and signaling, 629 genes (39.66%) were involved in metabolism, and 332 genes (20.93%) were poorly characterized. GraphPad Prism 8.0.3. (d) Classification of KEGG functions of *P. pentosaceus* XY62. A total of 1057 genes were annotated with specific functions, including 21 biological system functions, 45 cellular process functions, 103 environmental information processing functions, 154 genetic information processing functions, and 539 metabolic functions. GraphPad Prism 8.0.3.


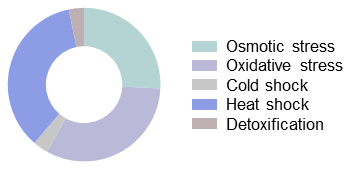

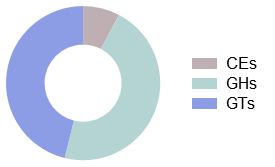
**Fig. S5**

**b**

**a**

**Supplementary Figure 5** Types of CAZymes and stress response genes in *P. pentosaceus* XY62. (a) Composition and abundance of CAZymes in *P. pentosaceus* XY62. GraphPad Prism 8.0.3. (b) Composition and abundance of stress response genes in *P. pentosaceus* XY62. GraphPad Prism 8.0.3.

**
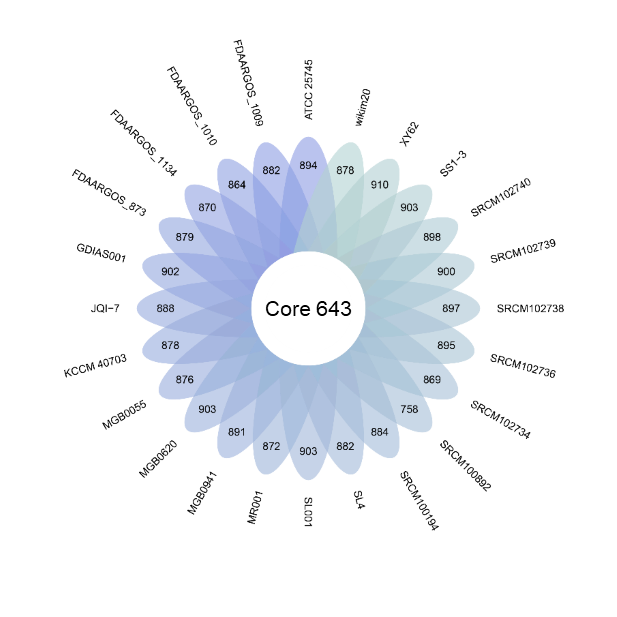

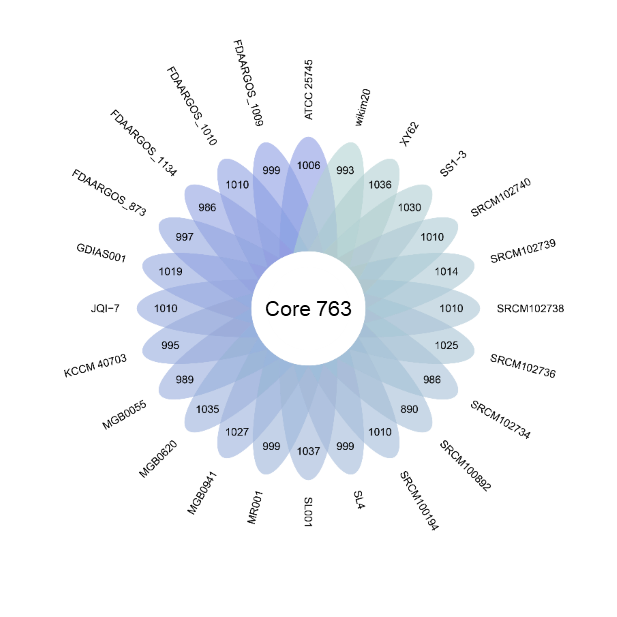

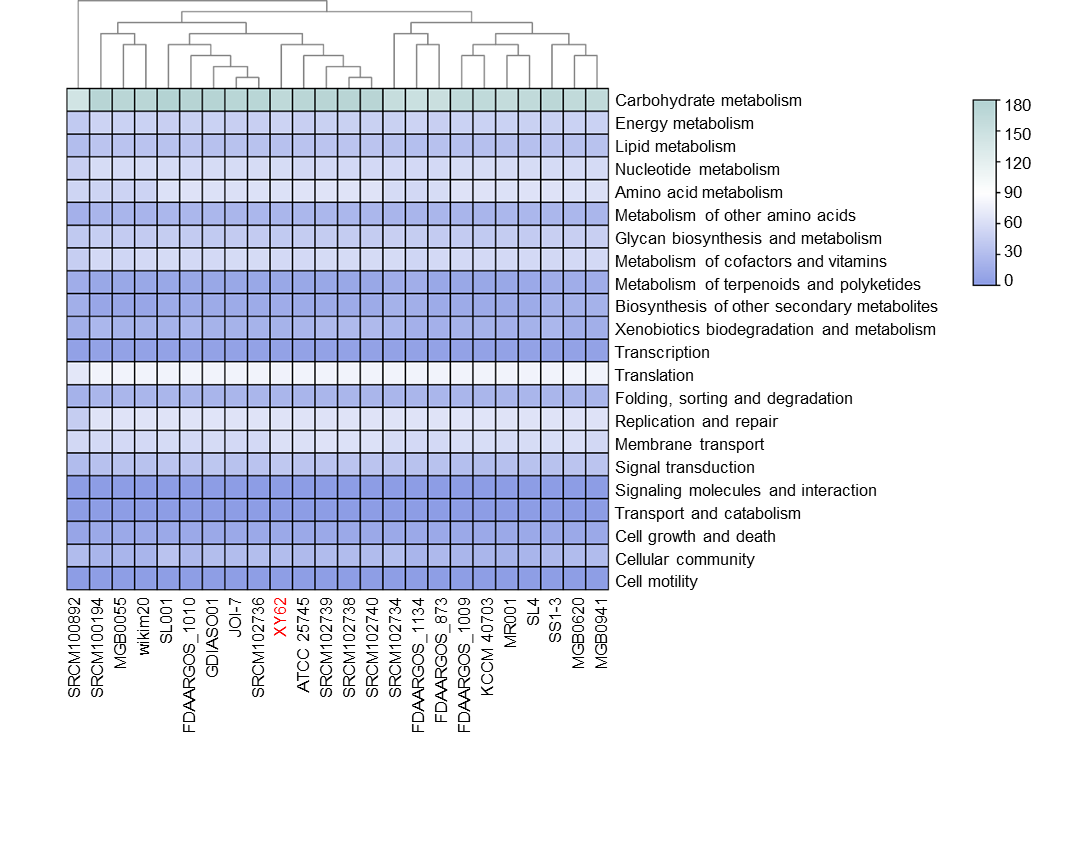
Fig. S6**

**c**

**b**

**a**

**Supplementary Figure 6** Differences in gene function among 24 strains. (a) The petal diagram shows the distribution of the COG gene in *P. pentosaceus* XY62 and the reference genomes. https://www.bic.ac.cn/BIC/#/. (b) The petal diagram shows the distribution of the KEGG gene in *P. pentosaceus* XY62 and the reference genomes. https://www.bic.ac.cn/BIC/#/. (c) Heat map of KEGG secondary functional differences among 24 strains. TBtools.

**Fig. S7**


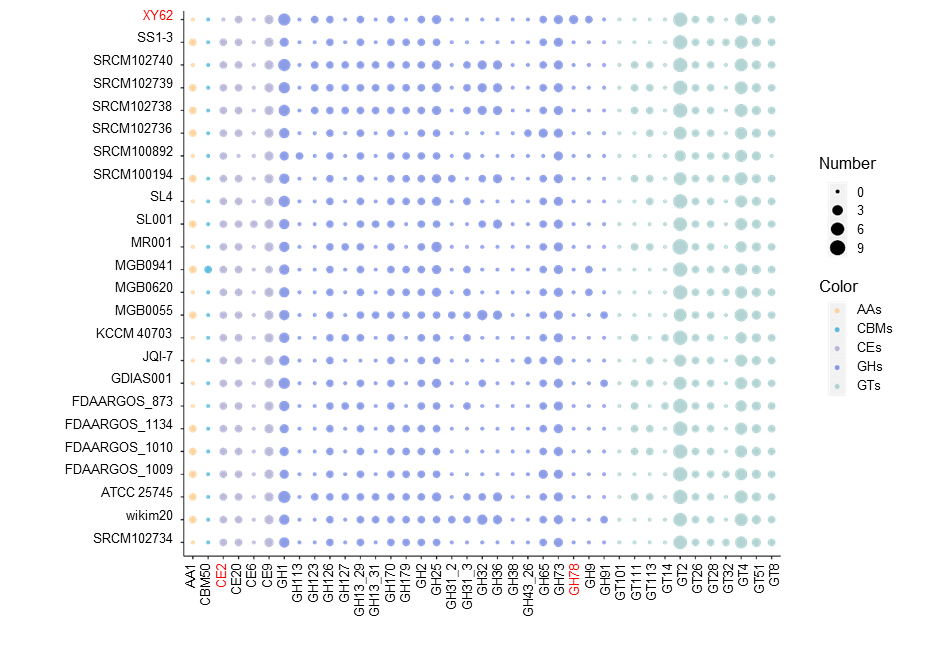


**Supplementary Figure 7** The variance in carbohydrate enzymes between *P. pentosaceus* XY62 and the reference genomes (https://www.bic.ac.cn/BIC/#/)

**Fig. S8**


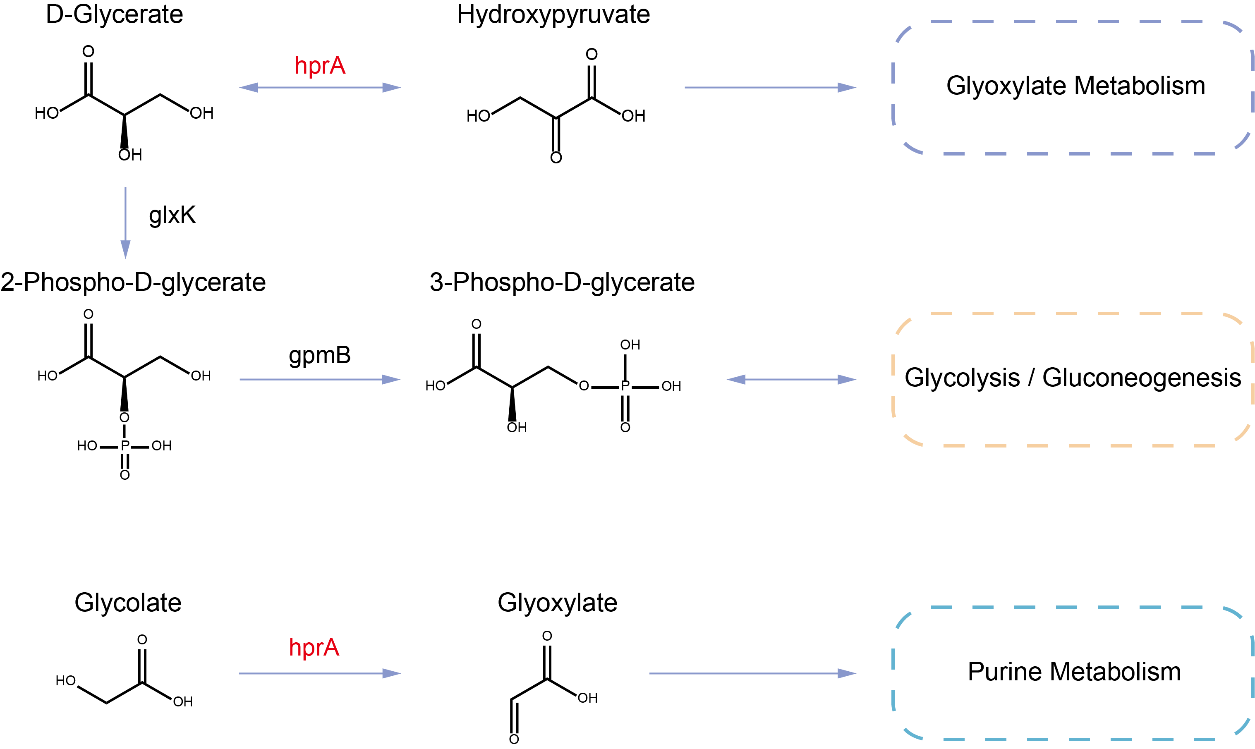


**Supplementary Figure 8** The metabolic pathway map of *hpr*A gene in *P. pentosaceus* XY62. Adobe Illustrator 2021.

**
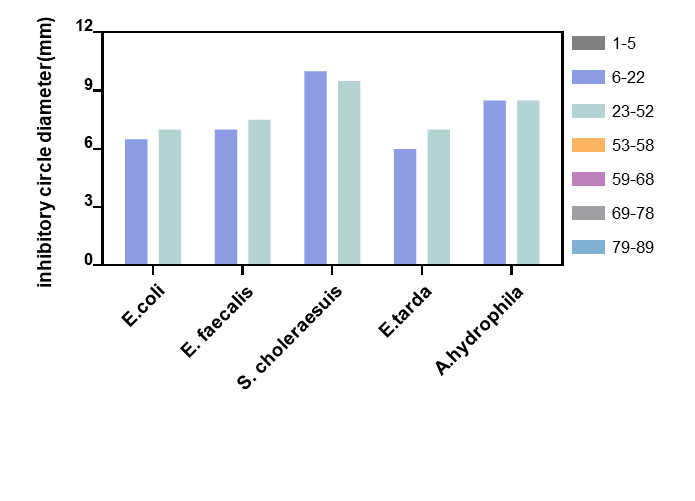

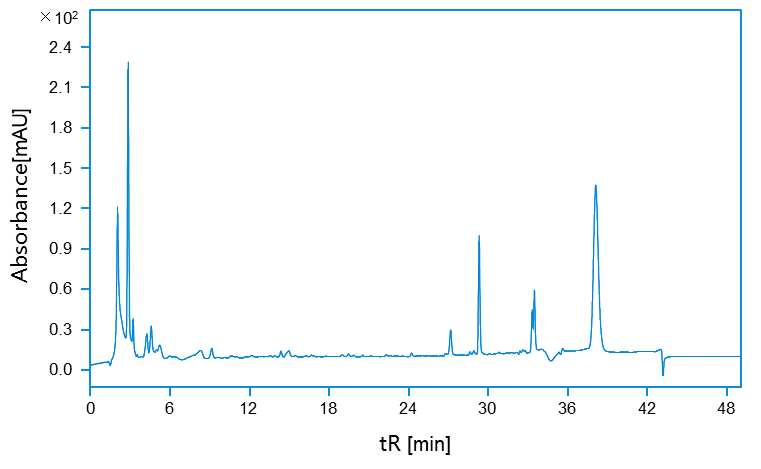

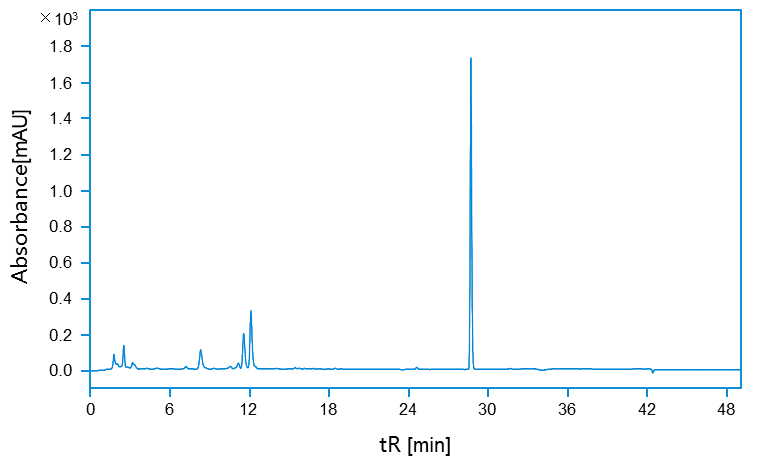
Fig. S9**

**c**

**b**

**a**

**Supplementary Figure 9** The UV absorption of metabolites from *P. pentosaceus* XY62 at a wavelength of 220 nm. (a) The UV absorption of component 2 at a wavelength of 220 nm. (b) The UV absorption of component 3 at a wavelength of 220 nm. (c) Antibacterial activity of seven crude components. GraphPad Prism 8.0.3.

**Fig. S10**

0

1.0

2.0

3.0

4.0

5.0

tR [min]

0.0

1.0

2.0

3.0

4.0

5.0

×10^2^

Absorbance[mAU]

Wavelength=220nm

**Supplementary Figure 10** The UV absorption of lactic acid at a wavelength of 220 nm.

**
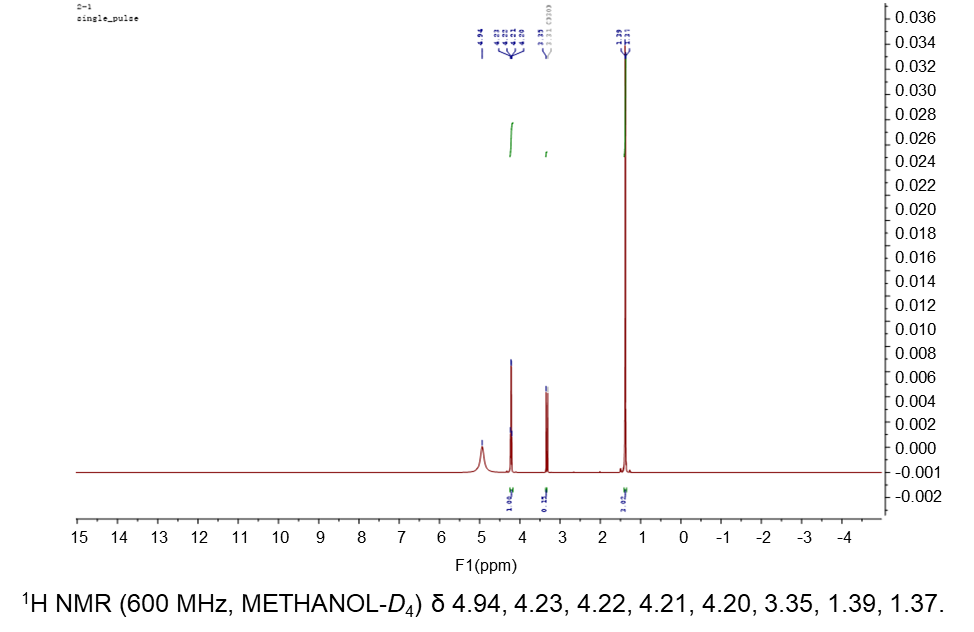

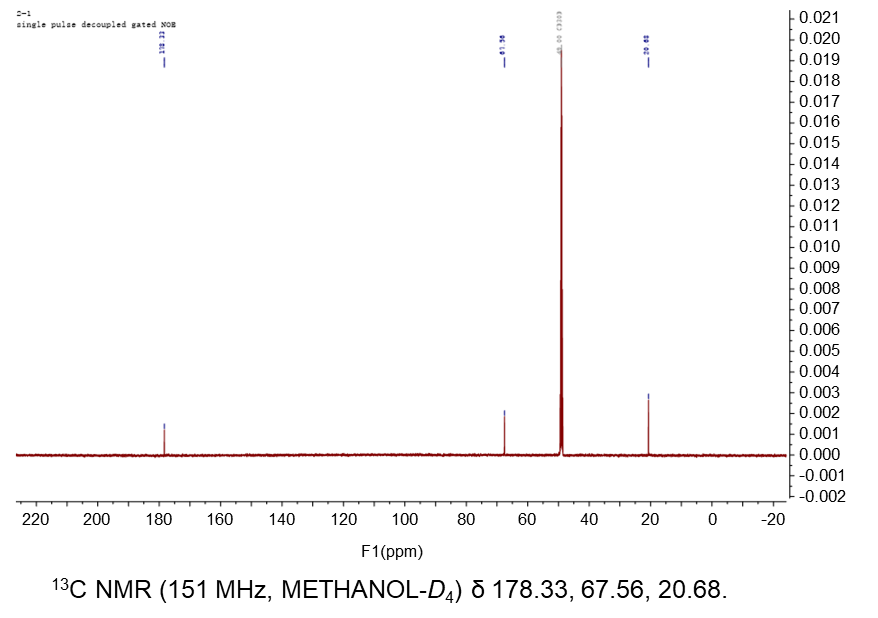
Fig. S11**

**b**

**a**

**Supplementary Figure 11** Nuclear magnetic resonance spectroscopy of lactic acid. (a) 1H-NMR spectrum of lactic acid in CD3OD. (b) 13C NMR spectrum of lactic acid in CD3OD.

**Fig. S12**

**a**

**
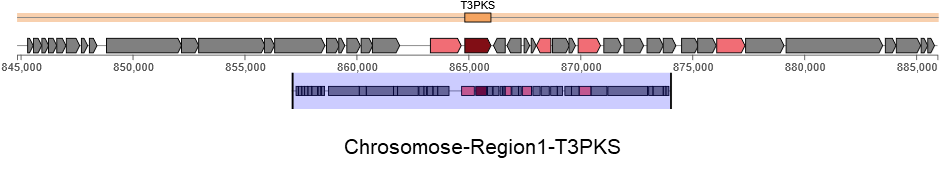
**


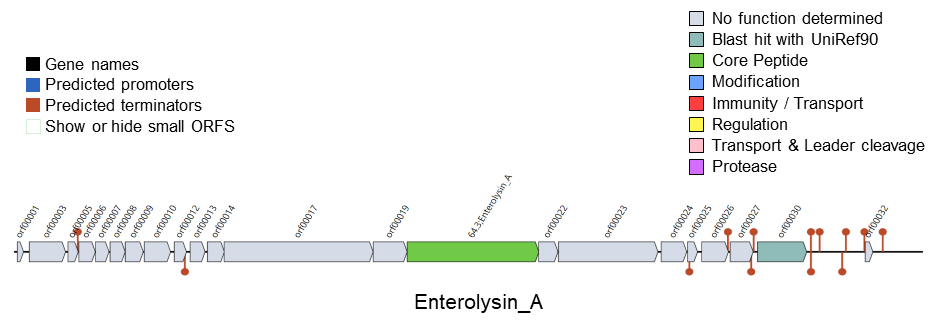


**b**

**Supplementary Figure 12** Metabolic gene clusters of *P. pentosaceus* XY62. (a) The type III polyketide synthases (T3PKS) gene clusters. https://antismash.secondarymetabolites.org/#!/start. (b) The Enterolysin A (En1A) gene clusters. http://bagel4.molgenrug.nl/.

**Table. S1**

**Supplementary Table 1** Formulation and pH Data of Media.

| Medium | Formulation | pH |
| --- | --- | --- |
| LB | Yeast extract 5 g/L, NaCl 5 g/L, Peptone 10 g/L, Agar 15 g/L | Natural pH |
| 2216E | Peptone 5 g/L, Yeast extract 1 g/L, Ferric citrate 0.1 g/L, NaCl 19.45 g/L, MgCl 5.98 g/L, Na_2_SO_4_ 3.24 g/L, CaCl_2_ 1.8 g/L, KCl 0.55 g/L, Na_2_CO_3_ 0.16 g/L, KBr 0.08 g/L, SrCl_2_ 0.034 g/L, H_3_BO_3_ 0.022 g/L, Na_2_SiO_3_ 0.004 g/L, NaF 0.0024 g/L,NH_4_NO_3_ 0.0016 g/L, Na_2_HPO_4_ 0.008 g/L | pH=7.6 |
| PDA | Potato 200 g/L, Glucose 20 g/L, AMP 0.1 g/L, Agar 15 g/L | Natural pH |
| YPD | Peptone 20 g/L, Yeast extract 10 g/L, Glucose 20 g/L, Agar 15 g/L | pH=6 |
| OMA | Oatmeal 30 g/L, Glucose 2 g/L, AMP 0.1 g/L, Agar 15 g/L | Natural pH |
| CMA | Maize flour 30 g/L, Glucose 2 g/L, AMP 0.1 g/L, Agar 15 g/L | Natural pH |
| BHIA | Peptone 10 g/LNa_2_PO_4_ 2.5 g/L, Beef Heart Infusion 17.5 g/L, NaCl 5 g/L, Glucose 2.0 g/L, Agar 15 g/L | pH=7.4 |
| NA | Tryptone 10 g/L, Beef extract 3g/L, NaCl 5g/L, Agar 15 g/L | Natural pH |
| Gauze's Synthetic Medium No.1 | Soluble starch 20 g/L, KNO_3_ 1g/L, K_2_HPO_4_ 0.5g/L, MgSO_4_.7H_2_O 0.5g/L, NaCl 0.5 g/L，FeSO_4_.7H_2_O 0.01 g/L, Agar 15 g/L | pH =7.2-7.4 |
| MM | Glucose 10 g/L, MgSO_4_.7H_2_O 0.2 g/L, FeSO_4_.7H_2_O 0.01 g/L, (NH_4_)_2_SO_4_ 1 g/L, K_2_HPO_4_ 0.5 g/L, Agar 15 g/L | Natural pH |
| YMG | Glucose 4g/L, Yeast extract 4g/L, Malt extract 10g/L, Agar 15 g/L | Natural pH |
| TSA | Glucose 40 g/L, Beef extract 2 g/L, Yeast extract 2 g/L，Peptone 6 g/L, Ammonium acetate 3 g/L, K_2_HPO_4_ 0.5g/L, MgSO_4_.7H_2_O 0.2 g/L, FeSO_4_.7H_2_O 0.01 g/L, Agar 15 g/L | pH=6.5 |
| RCM | Yeast extract 3 g/L, Beef extract 10 g/L, Peptone 10 g/L, Soluble starch 1 g/L, Glucose 5 g/L, L-Cysteine Hydrochloride 0.5 g/L, NaCl 3 g/L, NaAc 3 g/L, Agar 15 g/L | pH=8.5 |
| Shieh | Peptone 5 g/L, Yeast extract 0.5 g/L, CH_3_COONa.3H_2_O 0.01 g/L, BaCl_2_.2H_2_O 0.01 g/L, K_2_HPO_4_ 0.1 g/L, KH_2_PO_4_ 0.05 g/L, MgSO_4_.7H_2_O 0.3 g/L, CaCl_2_.2H_2_O 0.0067 g/L, FeSO_4_.7H_2_O 0.001g/L, NaHCO_3_ 0.05g/L, Agar 15 g/L | pH=7.2 |
| TSB | Casein Tryptone 15 g/L, Soya Peptone 5 g/L, NaCl 5 g/L, Beef extract 5 g/L, Agar 15 g/L | pH=7.2-7.4 |
| AM | (NH_4_) SO_4_ 5 g/L, K_2_HPO_4_ 2 g/L, MgSO_4_.7H_2_O 1 g/L，FeSO_4_.7H_2_O 0.01 g/L, Sodium alginate 5 g/L, Agar 15 g/L | pH=7.5 |
| MRS | Peptone 10 g/L, Beef extract 10 g/L, Yeast extract 5 g/L, Glucose 20 g/L, NaAc 5 g/L, Ammonium citrate dibasic 2 g/L, Tween -80 1 ml/L, K_2_HPO_4_ 2.0 g/L, MgSO_4_.7H_2_O 0.58 g/L, MnSO_4_.7H_2_O 0.25 g/L, Agar 15 g/L | pH=6.2-6.4 |
| Lignin Screening Medium | Lignin 2 g/L, NaCl 26 g/L, MgCl_2_·6H_2_O 5 g/L, CaCl_2_·2H_2_O 1.4 g/L, Na_2_SO_4_ 4 g/L, NH_4_Cl 0.3 g/L, KH_2_PO_4_ 0.1 g/L, KCl 0.5 g/L, Glucose 5.4 g/L, N-acetylglucosamine 2 g/L, Vitamin mixture 1 mL/L, Vitamin B_1_ 1 mL/L, Vitamin B_12_ 1 mL/L, NaHCO_3_ 0.0084 g/L, Aniline blue 0.05 g/L, Agar 15 g/L | pH=7.0 |
| BCP | K_2_HPO_4_ 1 g/L, MgSO_4_·7H_2_O 0.5 g/L, KCl 0.5 g/L, FeSO_4_ 0.01g/L, (NH_4_)_2_SO_4_ 2 g/L, Olive oil emulsion 120 mL/L, Agar 15 g/L, 5,5^,^ - Dibromo-o-cresolsulfonphthalein 4 mL/L | Natural pH |
| Cellulose Cong Red Medium | CMC-Na 10 g/L, KNO_3_ 1g/L, K_2_HPO_4_ 0.5 g/L, MgSO_4_.7H_2_O 0.5 g/L, FeSO_4_.7H_2_O 0.01g/L, NaCl 0.5 g/L, Agar 15 g/L | Natural pH |
| CZA | NaNO_3_ 3 g/L, K_2_HPO_4_ 1 g/L, MgSO_4_.7H_2_O 0.5 g/L, KCl 0.5 g/L, FeSO_4_ 0.01 g/L, Sucrose 30 g/L, Agar 15 g/L | Natural pH |
| Postgate Medium | Na_2_SO_4_ 3 g/L, KH_2_PO_4_ 0.3 g/L, K_2_HPO_4_ 0.5 g/L，NH_4_Cl 1 g/L, CaCl_2_ 0.1 g/L, Yeast extract 1 g/L, Sodium citrate dihydrate 0.3 g/L, Sodium lactate 6mL/L, MgSO_4_.7H_2_O 0.1 g/L, (NH_4_)SO_4_ 0.2 g/L, Vitamin C 0.1 g/L | Natural pH |
| MEA | Malt Extract 30 g/L, Peptone 3 g/L, Agar 15 g/L | Natural pH |

**Table. S2**

**Supplementary Table 2** Analysis of 16S rDNA Similarity in Seven Screened *Pediococcus pentosaceus* Strains.

| Number | Top-hit taxon | Top-hit strain | Similarity (%) | Isolation medium | Completeness (%) |
| --- | --- | --- | --- | --- | --- |
| XY62 | *P．pentosaceus* | DSM 20336 | 99.79 | YPD | 96.7 |
| XT501 | *P．pentosaceus* | DSM 20336 | 99.73 | TSA | 97.5 |
| XTO611 | *P．pentosaceus* | DSM 20336 | 99.72 | TSA | 96.2 |
| XR511 | *P．pentosaceus* | DSM 20336 | 99.79 | RCM | 96.7 |
| XMRS231 | *P．pentosaceus* | DSM 20336 | 99.86 | MRS | 96.3 |
| XM222 | *P．pentosaceus* | DSM 20336 | 99.72 | OMA | 96.7 |
| XM225 | *P．pentosaceus* | DSM 20336 | 99.73 | OMA | 97.5 |

**Table. S3**

**Supplementary Table 3** Genomic information of 24 *Pediococcus pentosaceus* strains for comparative genomic analysis.

| Strains | Source | Genome Sizes (Mb) | GC (%) | Plasmid | Protein-coding genes | RefSeq |
| --- | --- | --- | --- | --- | --- | --- |
| ATCC 25745 | Plant | 1.8 Mb | 37 | 0 | 1,728 | GCF_000014505.1 |
| FDAARGOS_873 | Food | 1.7 Mb | 37 | 0 | 1,673 | GCF_016028695.1 |
| FDAARGOS_1009 | Food | 1.8 Mb | 37 | 1 | 1,631 | GCF_016127775.1 |
| FDAARGOS_1010 | Food | 1.9 Mb | 37 | 5 | 1,855 | GCF_016128215.1 |
| FDAARGOS_1134 | Food | 1.7 Mb | 37 | 1 | 1,649 | GCF_016726885.1 |
| GDIAS001 | tapioca | 1.8 Mb | 37 | 0 | 1,763 | GCF_009791435.1 |
| JQI-7 | Fermented dairy | 1.7 Mb | 37 | 0 | 1,662 | GCF_006770865.1 |
| KCCM 40703 | sake mash | 1.8 Mb | 37 | 0 | 1,692 | GCF_002982155.1 |
| MGB0055 | kimchi | 1.8 Mb | 37 | 2 | 1,688 | GCF_023277765.1 |
| MGB0620 | kimchi | 2.1 Mb | 37 | 5 | 1,947 | GCF_023277805.1 |
| MGB0941 | kimchi | 2 Mb | 37 | 4 | 1,845 | GCF_023277785.1 |
| MR001 | Intestinal shrimp | 1.8 Mb | 37 | 0 | 1,757 | GCF_019614475.1 |
| SL001 | soil | 1.9 Mb | 37 | 1 | 1,826 | GCF_007923185.1 |
| SL4 | kimchi | 1.8 Mb | 37 | 0 | 1,716 | GCF_000496265.1 |
| SRCM100194 | Food | 1.9 Mb | 37 | 2 | 1,754 | GCF_002202155.1 |
| SRCM100892 | Food | 2 Mb | 37 | 6 | 1,593 | GCF_002173535.1 |
| SRCM102734 | Doenjang | 1.7 Mb | 37 | 0 | 1,607 | GCF_009930955.1 |
| SRCM102736 | Soybean paste | 1.8 Mb | 37 | 1 | 1,725 | GCF_009913955.1 |
| SRCM102738 | Soybean paste | 1.9 Mb | 37 | 1 | 1,770 | GCF_009913995.1 |
| SRCM102739 | Soybean paste | 1.9 Mb | 37 | 2 | 1,791 | GCF_009914015.1 |
| SRCM102740 | Soybean paste | 1.9 Mb | 37 | 1 | 1,765 | GCF_009914035.1 |
| SS1-3 | Adult feces | 1.8 Mb | 37 | 2 | 1,743 | GCF_003429405.1 |
| wikim20 | kimchi | 1.8 Mb | 37 | 3 | 1,703 | GCF_001411765.2 |
| XY62 | Mariana Trench Amphipodal gut | 1.8 Mb | 37 | 2 | 1882 | / |

**Table. S4**

**Supplementary Table 4** Types and quantities of drug resistance genes in the genome of *P. pentosaceus* XY62.

| Drug Class | Gene No. |
| --- | --- |
| macrolide antibiotic | 29 |
| tetracycline antibiotic | 18 |
| fluoroquinolone antibiotic | 13 |
| phenicol antibiotic | 10 |
| lincosamide antibiotic | 9 |
| streptogramin antibiotic | 8 |
| oxazolidinone antibiotic | 8 |
| acridine dye | 7 |
| glycopeptide antibiotic | 7 |
| pleuromutilin antibiotic | 6 |
| aminoglycoside antibiotic | 6 |
| aminocoumarin antibiotic | 6 |
| rifamycin antibiotic | 6 |
| peptide antibiotic | 5 |
| penam | 5 |
| mupirocin | 4 |
| diaminopyrimidine antibiotic | 4 |
| nitroimidazole antibiotic | 3 |
| cephalosporin | 2 |
| fosfomycin | 2 |
| carbapenem | 1 |
| monobactam | 1 |
| cephamycin | 1 |
| penem | 1 |
| isoniazid | 1 |
